# The Effect of Structure Building Small Mammals in a Shifting Arctic Landscape

**DOI:** 10.1002/ece3.71523

**Published:** 2025-06-04

**Authors:** Austin Roy, Jennie R. McLaren

**Affiliations:** ^1^ Department of Biology University of Texas at El Paso El Paso Texas USA

**Keywords:** herbivore, lemming, polygonal tundra, soil, vegetation community, zoogeochemistry

## Abstract

Landscapes are undergoing ecological changes, and how organisms interact with changing habitats has implications for zoogeochemical influences on ecosystem function (processes and properties). This may be especially true for organisms that alter nutrient cycling, such as structure builders, in nutrient‐limited systems such as the Arctic. Our aims were to examine the impact of brown lemming ( *Lemmus trimucronatus* ) structures (winter nests, runways, latrines, and burrows) on above‐ and below‐ground processes in habitats that represent contemporary (flat‐centered polygon) and potential future (high‐centered polygon) tundra conditions near Utqiaġvik, Alaska. Above‐ground, structures influenced vegetation community structure at local scales, with winter nests having lower moss and forb cover and burrows, latrines, and runways having lower litter and higher bare ground cover; although effects did not vary by habitat type. Furthermore, structures influenced plant diversity, which may be driven by structure types supporting unique plant species. Below‐ground, burrows had broad effects on soil nutrients, with soils under burrows generally having lower carbon and nitrogen contents and exo‐enzyme activities in both habitat types. Other structures increased nutrient availability, with soils under winter nests having higher ammonium concentrations compared to controls in both habitat types, whereas soils under latrines had higher phosphate and extractable organic carbon than controls, but only in high‐centered polygon tundra. Additionally, soil temperatures under winter nests were lower than at control sites, but only in flat‐centered polygon tundra, and soil pH was higher under winter nests and runways in both habitat types. Effects of structures on soil physical properties probably helped to regulate the effects of structures on soil nutrient availability. Finally, differences in structure effects between habitat types suggest that as high‐centered polygonal tundra becomes more prevalent, this herbivore's influence on ecosystem processes at local scales may feedback to alter the function of future arctic ecosystems.

## Graphical abstract

We examined the effects of structure builders on biogeochemical cycling under future arctic tundra conditions. We found that structures influence nutrient availability and vegetation communities differently between contemporary and future conditions. These effects suggest that structure builder effects at local scales will feedback to alter the function of future arctic ecosystems.

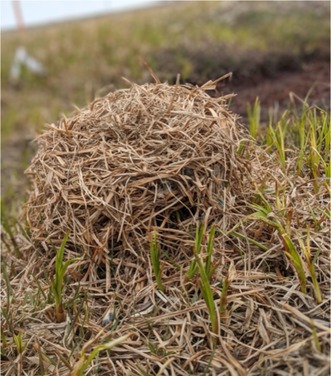

## 1 Introduction

Climate change is causing alterations to abiotic components of landscapes and ecosystems such as changes in hydrology, freeze–thaw cycles, and microtopography, resulting in effects on ecosystem function (Hodkinson et al. 1999; Lara et al. 2015; Wrona et al. 2016). Hand in hand with abiotic changes, biotic factors such as vegetation diversity and structure (Sistla et al. 2013; Lara et al. 2017), trophic and species interactions (Harrington et al. 1999), and individual species biology (Selwood et al. 2015), among others, are changing both directly and indirectly in response to climate change. Alterations in the abiotic and biotic components of a species' habitat are likely to change wildlife distributions and how species interact with their habitats (Sharma et al. 2009; Morris and Dupuch 2012; Baltensperger and Huettmann 2015; Sokolova et al. 2024). As wildlife are key components of ecosystems, changing wildlife interactions with these altered habitats may feedback to ecosystem function (processes and properties).

**FIGURE 1:**
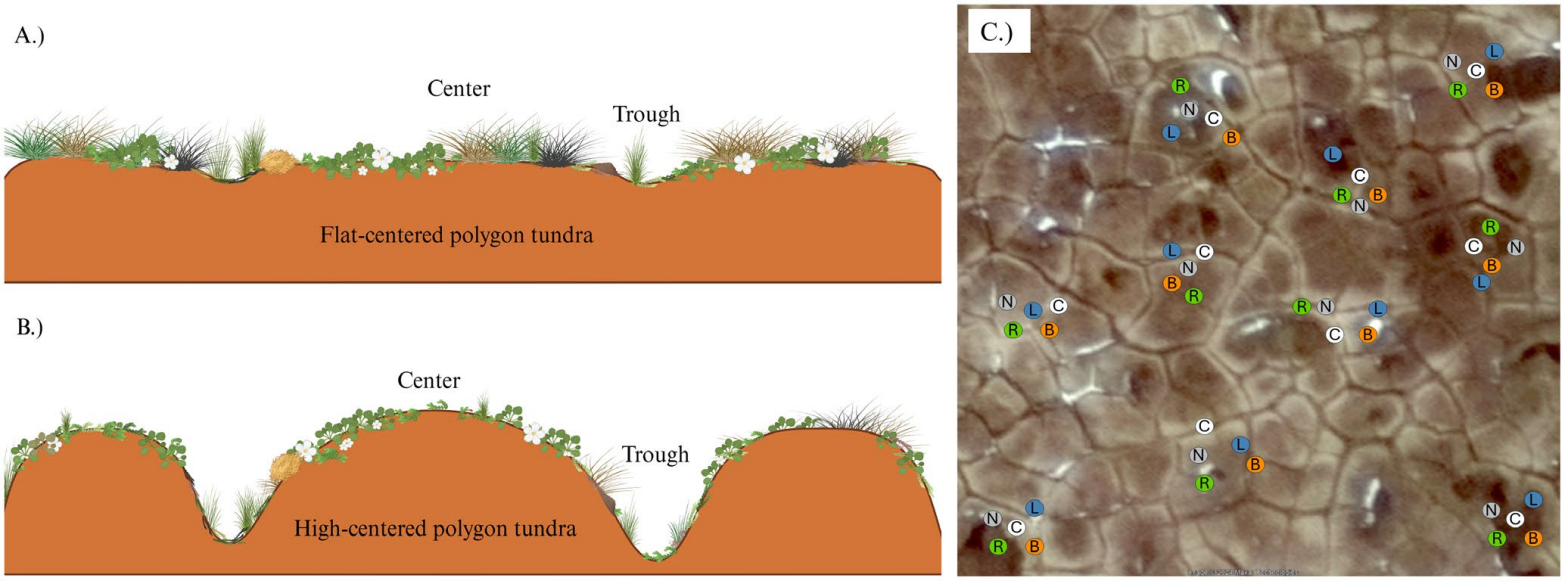
Diagrams depicting topographic and vegetation differences between (a) flat‐center polygon and (b) high‐center polygon tundra habitats, and (c) an example schematic diagram of sample locations within one habitat type near Utqiaġvik, AK, USA. Letters represent lemming structure types (nests [N], latrines [L], runways [R], burrows [B]) and control (C) sample sites.

Polygonal tundra is a common ecosystem type within many lowland arctic ecosystems (Nitzbon et al. 2019) making up an important fraction of thermokarst ecosystems, which cover up to 20% of the permafrost region in the Northern Hemisphere (Olefeldt et al. 2016), and up to 50% of tundra ecosystems in coastal Northern Alaska (Britton 1957; Hinkel et al. 2005). Polygonal tundra has developed over long periods due to seasonal freezing and thawing of tundra soils to form topographic polygons with low or flat centers (Hinkel et al. 2005). Increases in permafrost thawing lead to the degradation of this polygonal tundra, as expressed through the transition from low‐ or flat‐centered polygons to high‐centered polygons (Figure 1, French 2007; Liljedahl et al. 2012, 2016). These shifts to higher and more fragmented topography influence ecosystem properties such as hydrology, resulting in reduced inundation and increased runoff (Liljedahl et al. 2016), and changes in vegetation communities, where species abundances vary between the low‐ and high‐centered polygon tundra (Webber 1978). Such changes in ecosystem properties are linked to impacts on ecosystem processes such as carbon (C) cycling (Lara et al. 2015). A shift towards high‐center polygon tundra systems is occurring over relatively large areas and at a rapid pace (Liljedahl et al. 2012, 2016), leading to high‐centered polygon tundra representing future conditions across polygonal tundra types. Changes in these landscapes are having important impacts on human infrastructure (Nyland et al. 2017; Streletskiy et al. 2023) and ecosystem function (Lara et al. 2015). These effects on ecosystem processes may be intensified when paired with changes in wildlife use in arctic tundra and feedback to influence ecosystem function.

Within arctic ecosystems, small mammal herbivores are important regulators of ecosystem function through their zoogeochemical influences on the composition and physical structure of plant communities (Gough and Johnson 2018; Min et al. 2023), soil processes and nutrient availability (Stark et al. 2015; Roy et al. 2022), and feedback effects on C‐cycling (Tuomi et al. 2019; Min et al. 2021, 2023). An often‐overlooked aspect of small mammals' influence is their role as structure builders and ability to alter habitats (e.g., trample and clear vegetation and litter; Van der Wal et al. 2001; Hobbs and Searle 2005; Egelkraut et al. 2020) and conditions (e.g., soil moisture; Beylich et al. 2010) that would not occur without structure building. Some structures may concentrate or remove litter at given localities, while other structures may alter the vegetation community or remove vegetation cover that would be present otherwise and influence which plants are represented in the litter pool, and some structures may compact or loosen soils, with these effects resulting in influences on ecosystem processes via controls on nutrient inputs and cycling rates. Through structure building, small mammals can regulate biogeochemical cycling by increasing carbon, nitrogen (N), and phosphorus (P) pools in soils and plants at structure sites (Roy et al. 2022). The effects of small mammal structures have a potential for long‐term and ecosystem‐level influence over biogeochemical processes (Roy et al. 2022). Though small mammal structures (e.g., winter nests, runways, latrines, and burrows) seem to have significant roles in contemporary tundra ecosystem function, it is unclear whether these structures will continue to have similar effects in the future or in altered ecosystems. Through a better understanding of how small mammal structures will impact tundra systems under different ecosystem conditions, researchers will be better able to predict the future of ecosystem function in the Arctic.

The goal of this study was to examine whether the role of small mammal structures in arctic ecosystem function may be altered under future tundra conditions. Our specific aims were to determine how brown lemming ( *Lemmus trimucronatus* ) structures may alter vegetation communities and soil biogeochemical cycling at two sites representing contemporary and potential future tundra conditions.

## 2 Materials and Methods

### 2.1 Study Site

We conducted this study within the coastal tundra ecosystem located near Utqiaġvik, Alaska, during the peak of the growing season, when plant species could most accurately be identified, in the summer of 2021. This ecosystem contains eight tundra features (Webber et al. 1980), with polygonal tundra being a main tundra type on the landscape. The Utqiaġvik area has experienced rapid changes in the structure of polygonal tundra in recent decades (Lara et al. 2015). For this study, we sampled within one site containing flat‐centered polygon habitat (71.2757, −156.6114), representing intact, contemporary polygonal tundra conditions, and one site containing developed high‐center polygon habitat (71.2569, −156.8616), representing degraded, future polygonal tundra conditions (Zheng et al. 2018; Abolt et al. 2019). Flat‐centered polygon and high‐centered polygon habitats contain similar vegetation species, with species presence varying between aquatic and semi‐aquatic polygon troughs and edges, dominated by graminoids, through the relatively dry zone of polygon centers, dominated by dwarf shrubs, mosses, and lichens (Johnson et al. 2011; Assmann et al. 2019). However, due to inherent topographic differences between flat‐centered and high‐centered polygons, vegetation species representation varies between habitat types (Table S1). Brown lemmings ( *L. trimucronatus* ) are the dominant small mammal herbivore in the area and occur in both flat‐centered polygon and high‐centered polygon tundra (Batzli et al. 1983). Collared lemmings ( *Dicrostonyx groenlandicus* ) also occur in the ecosystem and are more commonly found in high‐centered polygon habitats (Batzli et al. 1980, 1983). During periods of high brown lemming abundance, they become dominant in habitats shared with collared lemmings (Morris et al. 2000; Gruyer et al. 2008). During the course of this study, brown lemmings were relatively abundant, and no collared lemmings were captured or observed within the study sites (Steketee, pers. comm., Holt, pers. comm.).

### 2.2 Methods

#### 2.2.1 Sampling Design

**FIGURE 2:**
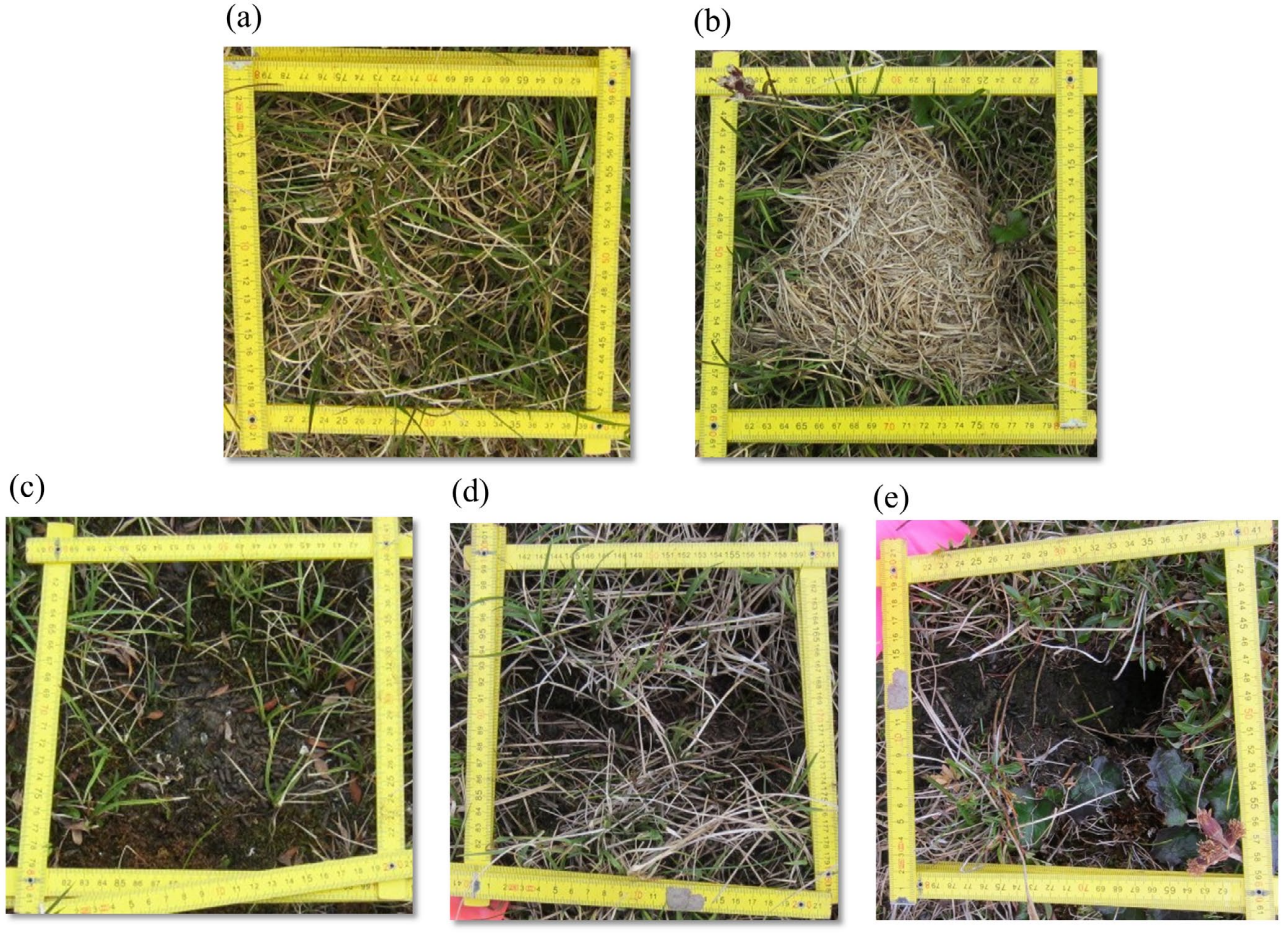
Examples of structures built by brown lemmings: (a) control plot, (b) winter nest, (c) latrine, (d) runway, and (e) burrow, and sampled within the study area located in arctic tundra near Utqiaġvik, AK, USA.

Within each habitat type (flat‐centered polygon, high‐centered polygon, Figure 1), we sampled from 10 areas located a minimum of 5 m apart, with distances between areas varying depending on the abundance of structures observed. Within each sample area, we sampled one of each structure type (winter nests, runways, latrines, burrow entrances [hereafter burrows]) and a control location (Figure 2a–e), with individual structures being at least 1 m apart; individual structure sites were no greater than 5 m apart. We only sampled at structures with fresh lemming sign (e.g., active runway, disturbed soil at burrows); however, as some structures can be used for multiple years (McKendrick et al. 1980), we did not assess structure age. Due to the amount of animal activity within some areas, we haphazardly selected control sites in areas 1–5 m from a structure, which lacked any visible small mammal activity and visually contained similar vegetation of non‐small mammal‐impacted tundra in the surrounding area. In total, we sampled 10 replicates of each structure type and 10 controls within each habitat type (Figure 1).

#### 2.2.2 Above‐Ground

##### 2.2.2.1 Vegetation Community

We assessed the vegetation community at each small mammal structure type and control location. We used 20 cm × 20 cm quadrats, which were centered on and typically encompassed the structure (Figure 2), to quantify the percentage cover of vascular and nonvascular plants, bare ground, and plant litter. This size quadrat was selected following previous sampling which found that a 40 × 40 cm quadrat was likely too broad to detect effects of structures (e.g., areas distant from a structure were unaffected and masked effects closer to the structure). We identified vascular plants to the species level, and we grouped mosses and lichens across species. We calculated relative cover within each plot for each species or cover class to standardize data across plots. We then conducted cover analyses on plant growth forms (graminoids, evergreen shrubs, deciduous shrubs, forbs, lichens, mosses, and fungi). Using the package vegan (Oksanen et al. 2022), in program R (v4.1.3, R Core Team 2023), we calculated species richness and Shannon diversity indices at each structure type and control at each site only using vascular plant species. We also collected bare ground and litter cover data at each plot.

##### 2.2.2.2 NDVI

At each sample site, we used a RapidScan (Model CS‐45, Holland Scientific) to measure normalized difference vegetation indices (NDVI). We collected four measurements per plot, approximately 0.5 m above and centered on the structure, by rotating the RapidScan 45° between each measurement. We then calculated mean NDVI for each sample site.

##### 2.2.2.3 Litter Height

At each structure location we collected litter height at a single point haphazardly located within the footprint of the structure (e.g., within the runway) or randomly inside of the cover quadrat for control plots using a ruler. We recorded litter height as the distance (cm) from the soil surface to the top of the litter layer.

#### 2.2.3 Below‐Ground

##### 2.2.3.1 Soil Nutrients

We collected soil samples under each structure and control location from the soil organic layer to a depth of 5 cm (approximately 5 × 5 × 5 cm) using a serrated bread knife. For each soil sample, we dried a subsample of known volume at 50°C for 48 h to assess bulk density and volumetric water content for calculations. Subsequently, we homogenized each remaining soil sample by hand, removing all large roots (> 1 mm diameter), and partitioned and froze samples for remaining analyses. We then shipped dried and frozen soil samples to the University of Texas at El Paso, where frozen samples were kept frozen at −20°C or −80°C (enzyme samples only) until analysis.

We analyzed dried soil samples for total C, N, and P and frozen samples for inorganic nutrients (${\mathrm{NH}}_{4}^{+}$, ${\mathrm{NO}}_{3}^{-}$, ${\mathrm{PO}}_{4}^{3-}$), total extractable nutrients (extractable organic C [EOC], extractable total N [ETN]), microbial biomass C, N, and P (MBC, MBN and MBP), and for extracellular enzyme activity (Roy et al. 2020, 2022).

We ground and processed dry soil subsamples for total C and N content using a dry combustion C and N analyzer (PyroCube, Elementar, Langenselbold, Germany). We determined total P content after ashing samples at 500°C, digesting using 6 M HCl, and then analyzing ${\mathrm{PO}}_{4}^{3-}$ content using a malachite green assay (D'Angelo et al. 2001). To determine soil inorganic nutrients, we thawed and extracted subsamples in 0.5 M K_2_SO_4_ and analyzed extractant using colorimetric microplate assays (BioTEK Synergy HT microplate reader, Winooski, Vermont, USA). We determined ${\mathrm{NH}}_{4}^{+}$‐N (${\mathrm{NH}}_{4}^{+}$) content using a modified Berlethot assay (Rhine et al. 1998), ${\mathrm{NO}}_{3}^{-}$‐N (${\mathrm{NO}}_{3}^{-}$) using a modified Griess assay (Doane and Horwath 2003), and ${\mathrm{PO}}_{4}^{3-}$‐P (${\mathrm{PO}}_{4}^{3-}$) using a malachite green assay (D'Angelo et al. 2001).

We determined EOC and ETN for the extracts mentioned above using an EOC/ETN analyzer (TOC‐V Series CN analyzer, Shimadzu Corporation, Kyoto, Japan). To determine microbial biomass C, N, and P, we conducted the above EOC and ETN assays on samples after a direct chloroform‐addition modification of the fumigation‐extraction method (Brookes et al. 1985; Voroney et al. 2006) prior to extraction. We calculated microbial biomass for C, N, and P (MBC, MBN, and MBP) by subtracting EOC, ETN, or ${\mathrm{PO}}_{4}^{3-}$, respectively, of non‐fumigated samples from that of fumigated samples.

We assessed extracellular enzyme (exoenzyme) activity for 10 exoenzymes involved in the microbial acquisition of C, N, and P (as in Roy et al. 2020): C‐acquiring enzymes (β‐glucosidase, β‐cellobiosidase, β‐xylosidase, α‐glucosidase), N‐acquiring enzymes (N‐acetyl‐glycosaminidase [NAG], leucine amino peptidase [LAP]) and P‐acquiring enzymes (phosphatase, phosphodiesterase), as well as the oxidative enzymes phenol oxidase and peroxidase. We blended 1 g of soil with a sodium acetate buffer to reflect natural soil conditions (pH = 4). We then incubated samples at 20°C and measured enzyme activity (fluorescence) every 30 min for 3.5 h following methods adapted from Saiya‐Cork et al. (2002) and McLaren et al. (2017). We performed oxidative enzyme analysis using an l‐3,4‐dihydroxyphenylalanine (l‐DOPA) substrate for phenol oxidase and peroxidase. Finally, we measured color absorbance at 460 nm using a reader after 24 h of incubation at 6°C.

##### 2.2.3.2 Soil Temperature

We recorded a single soil temperature within the upper organic layer at 2.5 cm depth for each structure and control site using a digital probe thermometer (Yard Mastery, FL, USA). For each habitat type, we collected data on the same day and at approximately the same time, before solar noon (1000–1200).

##### 2.2.3.3 pH and Conductivity

We measured soil pH and conductivity using a Thermo Scientific pH/conductivity meter (Elite PCTS) in soil solutions using soils from each sample location. We made soil solutions for each sample location by combining oven‐dried soil with DI water in a 1:15 w/v ratio when possible, although larger ratios were used for extremely organic soils where not enough mass of soil was present to reach a 1:15 ratio.

##### 2.2.3.4 Soil Respiration

We recorded soil respiration at each sample location using an EMG‐4 portable carbon dioxide gas analyzer (PP Systems, Amesbury, MA, USA). We removed vegetation to the soil surface from each sample location to reduce the influence of plant respiration on measurements.

#### 2.2.4 Statistical Methods

We performed statistical analysis using program R (v4.1.3, R Core Team 2023) with a cutoff of *p* < 0.05 for inferring statistical significance and 0.05 < *p* < 0.10 as non‐significant trends.

To test for differences in above‐ and below‐ground variables due to structure type (winter nest, runways, latrines, burrows, and controls) between habitat (flat‐centered polygon, high‐centered polygon), we constructed generalized linear mixed models (GLMMs) with a Gamma distribution and log link using the “glmer” function in package lme4 (Bates et al. 2015); when models did not converge we used an inverse link. Our fixed effects included habitat and structure type and the habitat × structure type interaction. Each structure sampled was treated as an individual replicate. To account for differences among sample areas, we included sample area within a habitat type as a random effect. Delta *R* ^2^ values for GLMMs were calculated using the “r.squaredGLMM” function within package MuMIn (Barton 2022). For functional cover only (graminoids, evergreen shrubs, deciduous shrubs, forbs, lichens, mosses, fungi, bare ground, and litter), we converted the data to proportions and used GLMs with a Beta distribution and logit link to assess differences due to structure type and habitat. For data points containing zero (0) or one (1), we adjusted the values by 0.0001 to meet requirements of a Beta distribution. We examined the significance of main effects and their interactions within the GLMMs and GLMs using the “Anova” function within package car (Fox and Weisberg 2019) to run Chi‐squared tests. We then ran post hoc pair‐wise comparisons using the “lsm” function within the emmeans package (Lenth et al. 2023) using a holm correction for multiple comparisons for all interactions. We calculated effect sizes by dividing the larger value by the smaller value (e.g., mean pH at latrine sites/mean pH at control sites).

## 3 Results

While there were multiple differences in above‐ and below‐ground variables between structure types (Table S2, Figures S1–S8), here we report comparisons of structures to controls in both habitats.

### 3.1 Above‐Ground

**FIGURE 3:**
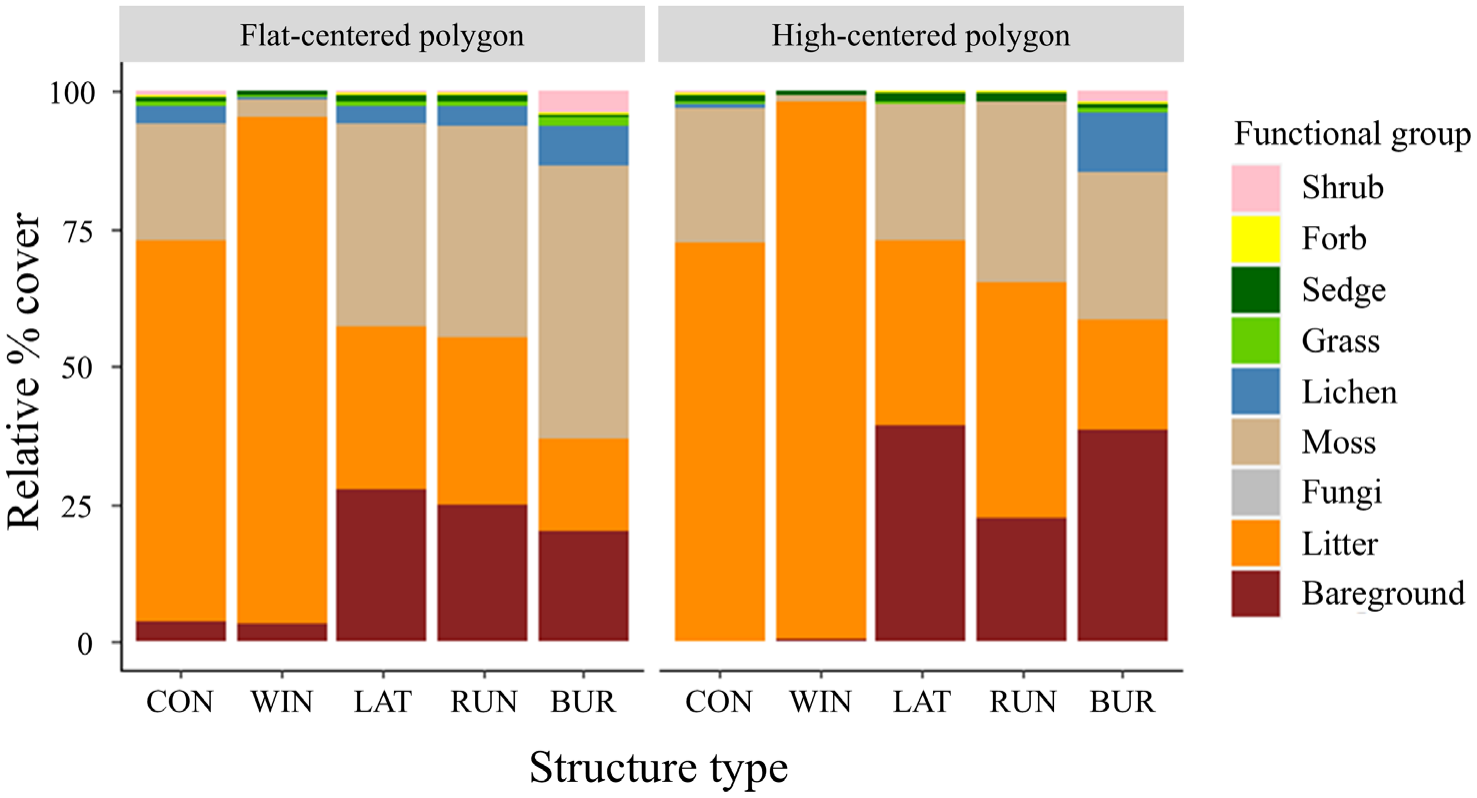
Stacked box plots showing relative percent cover of cover classes at control site locations (CON) and brown lemming structures (WIN = winter nest, LAT = latrine, RUN = runway, BUR = burrow) in flat centered polygon and high‐centered polygon tundra habitats. Ten replicates of each structure type and 10 control sites were sampled within each habitat type during the summer of 2021, near Utqiaġvik, Alaska, USA.

Vegetation Cover—We observed multiple differences in the relative cover of different vegetation growth forms due to habitat and structure type, without habitat × structure interactions (Table 1, Figures S1 and S2). Generally, shrub and grass cover was higher in flat‐centered tundra and forb and sedge were higher in high‐center polygon tundra (Table 1, Figures S1 and S2). We found that relative to control sites, winter nests had lower moss (*p* = 0.030) cover and a trend for lower forb (*p* < 0.084) cover (Figure 3). Burrows, runways, and latrines each had higher litter cover (*p* < 0.001) and lower bare ground (*p* < 0.001) than control locations.

**TABLE 1:** Results of GLMMs and Chi‐squared tests of brown lemming structures (S) and habitat type (H) on the relative cover of functional groups, plant diversity (Shannon index), plant species richness, litter height, and productivity (NDVI) within flat‐centered and high‐centered polygonal tundra habitat types.

Variable	Model *R* ^2^		Structure (*S*)		Habitat (*H*)		*S* × *H*	
	Marg	Cond	*χ* ^2^	*p*	*χ* ^2^	*p*	*χ* ^2^	*p*
Cover								
Shrub	0.10	0.10	2.71	0.609	5.42	0.020	0.95	0.917
Forb	0.24	0.24	**17.37**	**0.002**	**5.23**	**0.022**	1.71	0.788
Sedge	0.27	0.27	**14.23**	**0.007**	**10.01**	**0.002**	0.78	0.942
Grass	0.28	0.28	**14.03**	**0.007**	**9.81**	**0.002**	0.82	0.935
Fungi	0.01	0.01	1.15	0.886	0.08	0.784	0.28	0.991
Moss	0.23	0.23	**28.25**	**< 0.001**	0.93	0.335	3.77	0.437
Lichen	0.15	0.15	3.18	0.053	0.27	0.002	0.64	0.959
Litter	—	—	**104.76**	**< 0.001**	0.40	0.525	1.18	0.882
Bare ground	0.80	0.80	**52.48**	**< 0.001**	0.02	0.901	3.99	0.407
Shannon diversity	0.16	0.16	**14.61**	**0.006**	0.22	0.642	3.85	0.427
Species richness	0.18	0.20	**18.90**	**0.001**	0.65	0.421	3.81	0.432
Litter Depth	0.81	0.82	470.74	< 0.001	0.66	0.416	**18.31**	**0.001**
NDVI	0.13	0.13	*8.07*	*0.089*	**4.75**	**0.029**	1.34	0.854

*Note:* Degrees of freedom (df) for analyses were as follows: *S* (df = 4), *H* (df = 1), *S* × *H* (df = 4). Bolded text represents statistically significant results (*p* < 0.05) and italicized text represents non‐significant trends (0.05 < *p* < 0.10). Non‐bolded *p*‐values < 0.05 showed statistically non‐significant results during post hoc tests. Ten replicates of each structure type and 10 control sites were sampled within each habitat type during the summer of 2021, near Utqiaġvik, Alaska, USA.

#### 3.1.1 Vegetation Diversity

**FIGURE 4:**
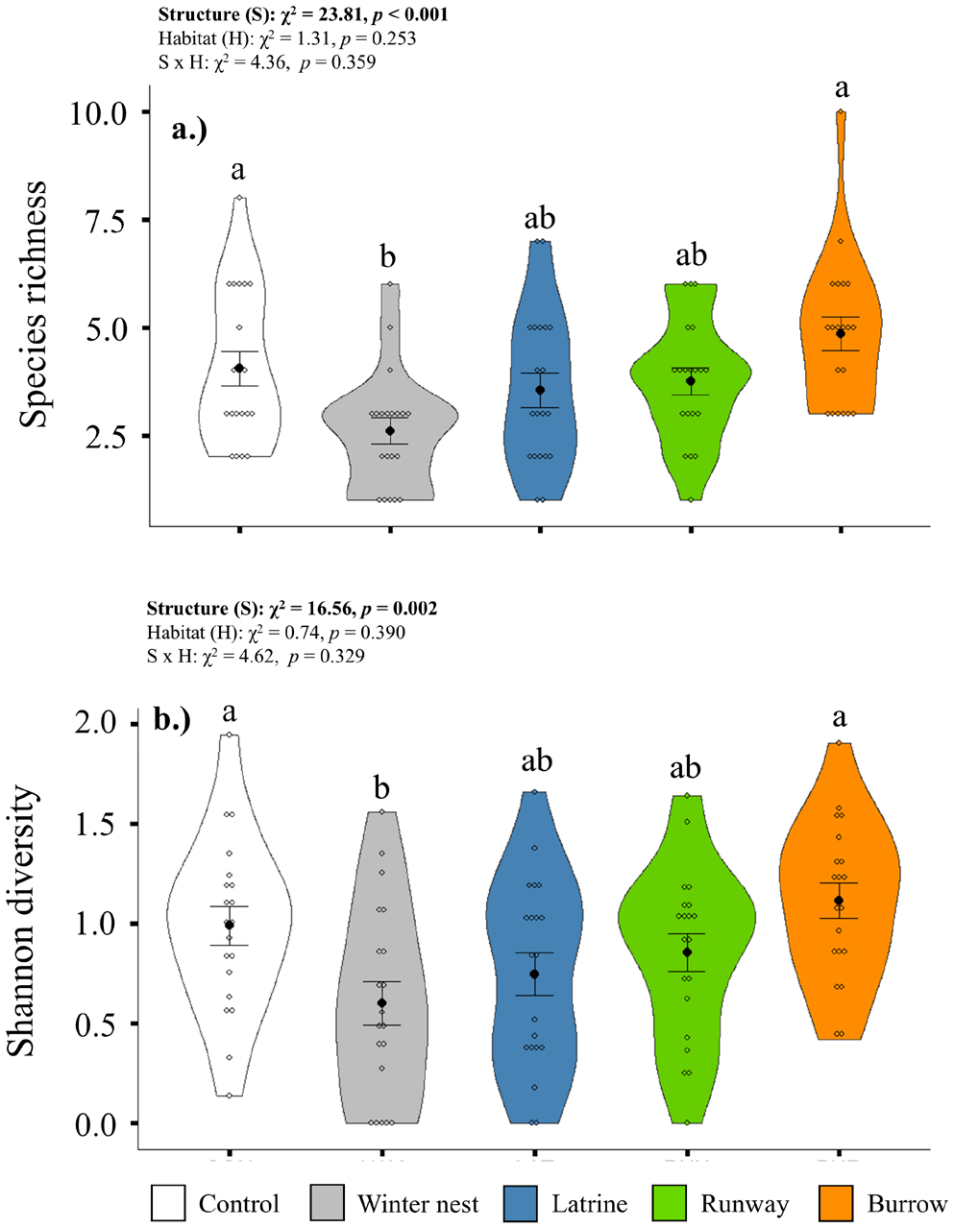
Violin plots showing differences, as determined through GLMMs, in vegetation species richness (a) and Shannon diversity indices (b) at brown lemming structures (WIN = winter nest, LAT = latrine, RUN = runway, BUR = burrow) and control site (CON) location. Open circles represent data points, black circles represent means, and error bars represent standard error. Ten replicates of each structure type and 10 control sites were sampled within each habitat type during the summer of 2021, near Utqiaġvik, Alaska, USA.

We observed a total of 23 vascular plant species at lemming structure sites and controls (Table S1). Seven species were unique to a habitat type (Table S1). Additionally, four species were unique to a specific structure type. *Luzula confusa* , *Pedicularis* spp., and *Potentilla hyparctica* were only detected at burrows, and *Polygonium* spp. was only detected at runway sites. Additionally, *Luzula arctica* was detected at all structure types, but not at control sites. For both species richness and Shannon diversity indices, effects varied by structure type, but not habitat, with no structure × habitat interaction (Table 1). Species richness was 1.6× lower at winter nest sites than controls (*p* = 0.042, Figure 4a), and similarly, Shannon diversity trended 1.5× lower at winter nest sites than control sites (*p* = 0.062, Figure 4b).

#### 3.1.2 NDVI

We detected differences in NDVI between habitat types, but not due to structure type, and with no interaction (Table 1). We acknowledge that including the structure in the measurement likely influenced our results; however, we were limited by the distance the meter needed to be above ground (0.5 m) and the potential spatial effect of each structure type (e.g., within 0.5 m of a structure).

#### 3.1.3 Litter Height

**FIGURE 5:**
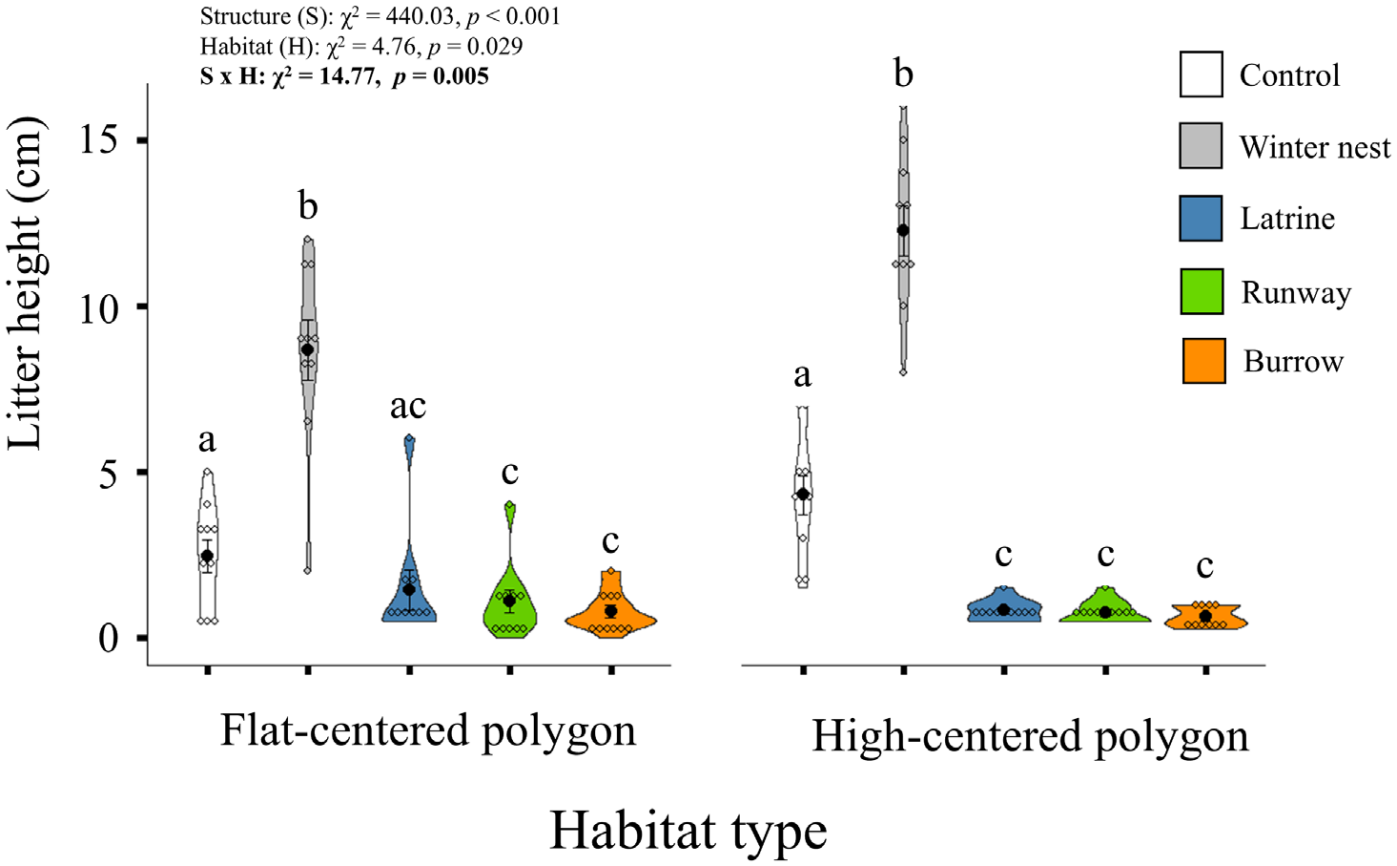
Violin plots showing differences, as determined through GLMMs, in litter height at control sites and brown lemming structures (winter nest, latrine, runway, burrow) in flat centered polygon and high‐centered polygon tundra habitats. Open circles represent data points, black circles represent means, and error bars represent standard error. Ten replicates of each structure type and 10 control sites were sampled within each habitat type during the summer of 2021, near Utqiaġvik, Alaska, USA.

We found significant differences in litter height with a habitat × structure interaction (Table 1). Specifically, we found that in flat‐centered polygon tundra, winter nests had 4.3× greater litter height than controls (*p* < 0.001), whereas burrows (*p* = 0.005) and runways (*p* = 0.014) had 2.6× and 2.5× lower litter height than controls, respectively (Figure 5). Responses were similar in the high‐centered polygon tundra, as winter nests had 12.3× greater litter height than controls (*p* < 0.001), and burrows (6.6×) and runways (5.7×) had lower litter height than control sites (*p* < 0.001) but in high‐centered polygon tundra, latrines also had lower (5.2×) litter height than control sites (*p* < 0.001).

### 3.2 Below‐Ground

#### 3.2.1 Soil Nutrients

**FIGURE 6:**
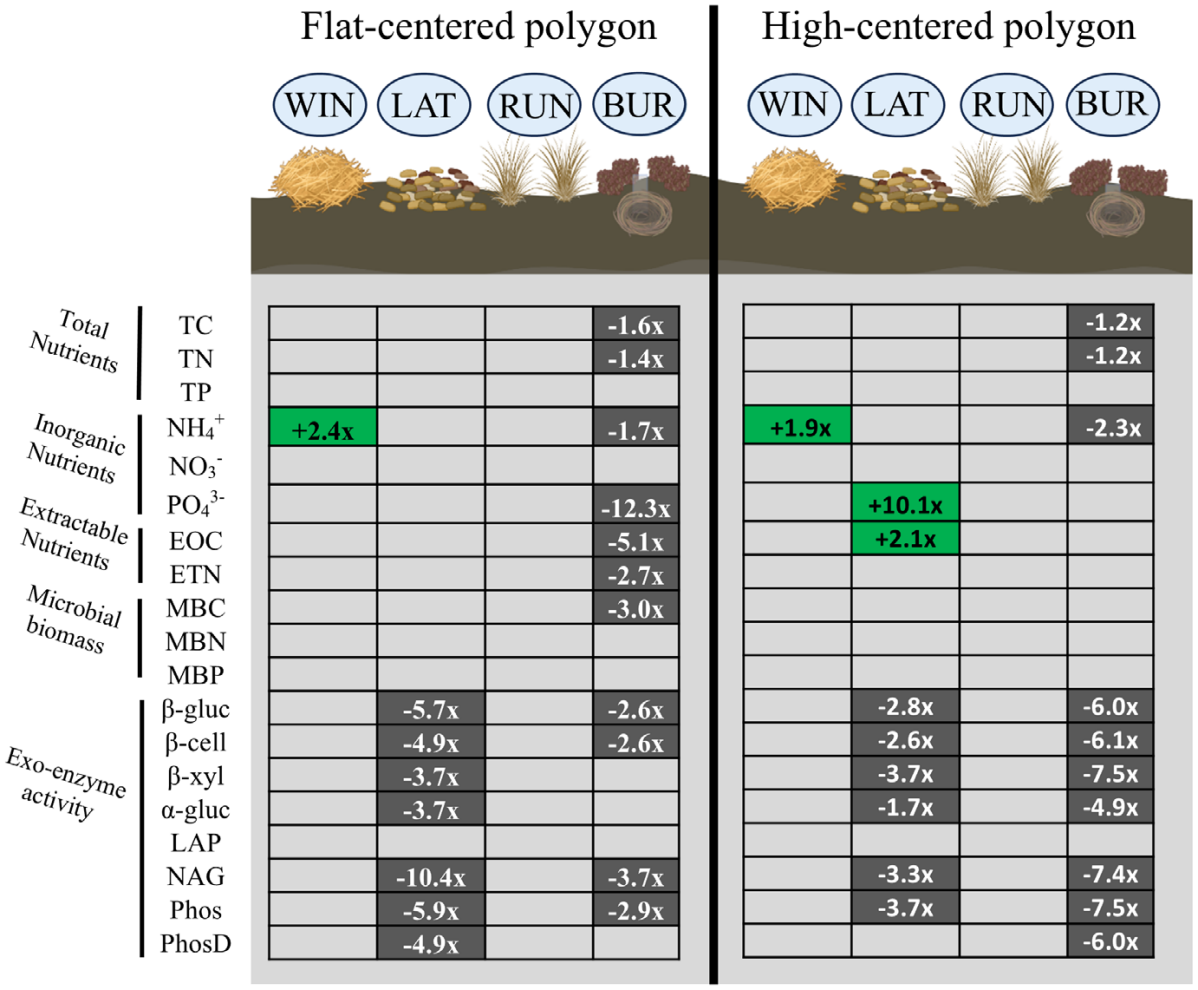
Summary of effects on nutrient pools in soils collected under brown lemming structures (WIN = winter nest, LAT = latrine, RUN = runway, BUR = burrow) compared to control (CON) locations in the flat‐centered polygon and high‐centered polygon tundra habitats. Numeric values represent effect sizes higher (+) or lower (−) compared to controls. Ten replicates of each structure type or controls from each habitat type were used in analyses. Green cells represent higher variables compared to controls, gray cells represent lower values compared to controls, and variables without numeric values and variables not shown did not have statistically significant effects compared to control sites. Effect sizes are based on mean values from each structure type and controls. Ten replicates of each structure type and 10 control sites were sampled within each habitat type during the summer of 2021, near Utqiaġvik, Alaska, USA.

We observed multiple effects of small mammal structures on soil nutrient pools within both habitat types (Table 2, Table S2). Burrows influenced the highest number of nutrient variables, generally having lower nutrient concentrations and enzyme activities compared to control sites (Figure 6). Latrines had variable effects depending on the habitat they were in, whereas winter nests affected few variables (Figure 6). Finally, runways had no statistical effects on soil nutrient variables.

**TABLE 2:** Results of GLMMs and Chi‐squared tests of brown lemming structures (S) and habitat type (H) on soil variables (total % carbon (C), nitrogen (N), and phosphorus (P) content (TC, TN, TP); ammonium (NH_4_); nitrate (${\mathrm{NO}}_{3}^{-}$); phosphate (${\mathrm{PO}}_{4}^{3-}$); extractable organic C (EOC); extractable total N (ETN); microbial biomass C, N, and P (MBC, MBN, MBP); eight hydrolytic enzymes (β‐glucosidase, β‐cellobiosidase, β‐xylosidase, α‐glucosidase, LAP, NAG, Phosphatase, Phosphodiesterase); two oxidative enzymes (Phenol oxidase, peroxidase)) within flat‐centered and high‐centered polygonal tundra habitat types.

Variable	Model *R* ^2^		Structure (*S*)		Habitat (*H*)		*S* × *H*	
	Marg	Cond	*χ* ^2^	*p*	*χ* ^2^	*p*	*χ* ^2^	*p*
TC	0.20	0.25	**17.95**	**0.001**	0.60	0.437	2.94	0.569
TN	0.14	0.24	**9.88**	**0.042**	0.10	0.755	2.10	0.718
TP	0.15	0.25	**11.50**	**0.021**	0.88	0.348	5.14	0.274
NO_3_	0.09	0.23	**11.41**	**0.022**	**4.33**	**0.038**	5.01	0.286
NH_4_	0.37	0.40	**38.07**	**< 0.001**	**14.33**	**< 0.001**	6.41	0.171
PO_4_	0.38	0.42	41.82	< 0.001	20.29	< 0.001	**18.44**	**0.001**
EOC	0.44	0.51	39.10	< 0.001	22.09	< 0.001	**31.53**	**< 0.001**
ETN	0.30	0.38	34.87	< 0.001	0.09	0.769	**13.44**	**0.009**
MBC	0.26	0.32	27.39	< 0.001	1.35	0.245	**13.08**	**0.011**
MBN	0.22	0.26	19.83	0.001	2.36	0.124	**10.50**	**0.033**
MBP	0.19	0.25	7.37	0.118	18.31	< 0.001	**9.61**	**0.048**
β‐glucosidase	0.57	0.59	109.62	< 0.001	0.44	0.507	**25.37**	**< 0.001**
β‐cellobiosidase	0.48	0.50	69.58	< 0.001	1.16	0.282	**20.22**	**< 0.001**
β‐xylosidase	0.59	0.60	113.94	< 0.001	3.42	0.064	**24.42**	**< 0.001**
α‐glucosidase	0.36	0.36	42.43	< 0.001	0.73	0.392	**10.93**	**0.027**
LAP	0.46	0.50	4.44	0.350	61.43	< 0.001	**23.43**	**< 0.001**
NAG	0.62	0.64	145.12	< 0.001	0.05	0.824	**28.96**	**< 0.001**
Phosphatase	0.72	0.72	170.15	< 0.001	0.04	0.839	**25.04**	**< 0.001**
Phosphodiesterase	0.59	0.60	104.91	< 0.001	5.03	0.025	**19.76**	**0.001**
Phenol oxidase	—	—	5.04	0.028	—	—	—	—
Peroxidase	—	—	9.15	0.058	—	—	—	—
Soil Temp	0.32	0.45	31.06	< 0.001	12.04	< 0.001	**14.18**	**0.007**
pH	0.12	0.34	**10.35**	**0.035**	0.01	0.927	6.68	0.154
Conductivity	0.37	0.47	18.30	0.001	25.61	< 0.001	**12.74**	**0.013**
Soil respiration	0.11	0.24	6.04	0.196	3.67	0.056	**12.10**	**0.017**

*Note:* Degrees of freedom (df) for analyses were as follows: *S* (df = 4), *H* (df = 1), *S* × *H* (df = 4). Bolded text represents statistically significant results (*p* < 0.05) and italicized text represents non‐significant trends (0.05 < *p* < 0.10). Non‐bolded *p*‐values < 0.05 showed statistically non‐significant results during post hoc tests. Ten replicates of each structure type and 10 control sites were sampled within each habitat type during the summer of 2021, near Utqiaġvik, Alaska, USA.

For C pools, we observed multiple structure, habitat, and structure × habitat interaction effects (Table 2). We found that total % C was lower under burrows compared to control sites in both habitat types (*p* = 0.007, Table 2, Figure 6, including effect sizes based on means). For EOC, burrows were lower than controls in flat‐centered polygon tundra (*p* < 0.001), but not high‐centered polygon, and latrines were higher than controls in high‐centered polygon (*p* = 0.036), but not flat‐centered polygon tundra (Figure 6). Additionally, MBC was lower under burrows (*p* = 0.004), but only in flat‐centered polygon habitats (Figure 6). C‐acquiring enzyme activities also showed multiple structure × habitat interactions (Table 2). β‐glucosidase and β‐cellobiosidase activities were lower under latrines and burrows than control sites in both habitat types (Figure 6, Table S2). β‐xylosidase was lower under latrines than controls in both sites but was only lower under burrows in high‐centered polygon habitat (Figure 6, Table S2). Additionally, α‐glucosidase activity was lower under latrines than controls in flat‐centered polygon habitat and lower under burrows than controls in high‐centered polygon habitat (Figure 6, Table S2).

For N pools, we observed that total % N was lower under burrows compared to control sites (Table 2, Figure 6). ${\mathrm{NH}}_{3}^{-}$ varied between sites and structure types (Table 2, Figure 6); however, there were no differences between structures and controls (*p* > 0.10). We found effects of structure type on ${\mathrm{NH}}_{4}^{+}$ (Table 2, Figure 6), with higher ${\mathrm{NH}}_{4}^{+}$ under winter nests (*p* = 0.011) and lower ${\mathrm{NH}}_{4}^{+}$ under burrows (*p* = 0.038) compared to controls. For ETN and NAG enzyme activity, we observed structure × habitat interactions (Table 2). ETN was lower under burrows compared to controls, but only in flat‐centered polygon habitat (*p* = 0.014). Additionally, NAG activity was lower under latrines and burrows, but in both habitats (Figure 6, Table S2).

For P pools, we observed several structure × habitat interactions (Table 2, Figure 6). We found that ${\mathrm{PO}}_{4}^{3-}$ was lower under burrows compared to controls in flat‐centered polygon habitat (*p* < 0.001, Figure 6), but higher under latrines than controls in high‐centered polygon habitat (*p* = 0.002, Figure 6). Phosphatase activity was lower under both latrines and burrows compared to controls (*p* < 0.001) within both habitat types (Figure 6, Table S2). Furthermore, Phosphodiesterase activity was lower under latrines in flat‐centered polygon habitat, but lower under burrows in high‐centered polygon habitat (Figure 6, Table S2).

#### 3.2.2 Soil Temperature

**FIGURE 7:**
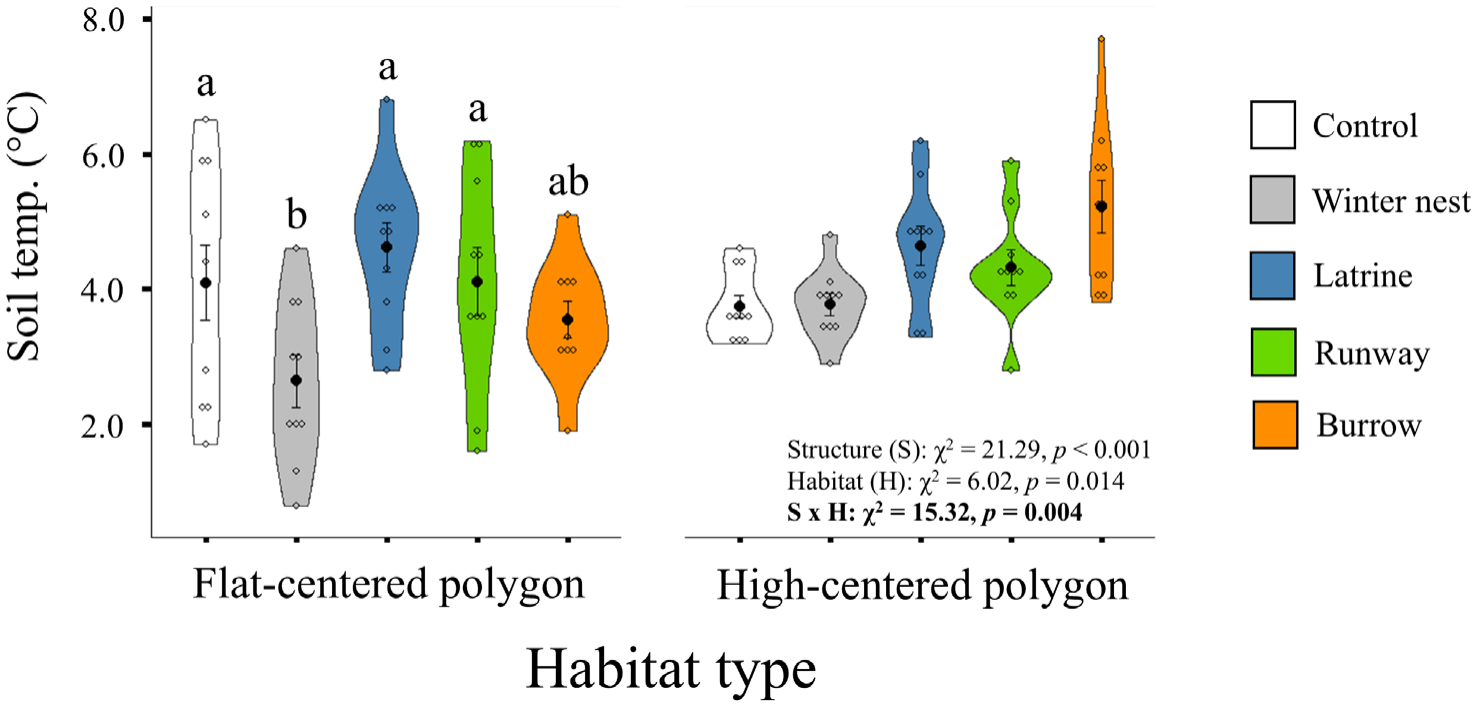
Violin plots showing differences, as determined through GLMMs, in soil temperatures at control site and brown lemming structure locations in flat‐centered polygon and high‐centered polygon tundra habitats. Soil temperature was recorded at 2.5 cm below the soil surface. Open circle represent data points, black circles represent means, and error bars represent standard error. Ten replicates of each structure type and 10 control sites were sampled within each habitat type during the summer of 2021, near Utqiaġvik, Alaska, USA.

We observed a structure × habitat interaction for soil temperature (Table 2). We found that soil temperatures under winter nests within flat‐centered polygon habitat were 1.5×, 1.8, and 1.6× lower than control sites (*p* = 0.003), latrines (*p* < 0.001), and runways (*p* = 0.002), respectively, but no temperature effects were seen due to structures in high‐centered polygon habitat (*p* > 0.100, Figure 7).

#### 3.2.3 pH

Soil pH varied by structure type, but not habitat type, with no interaction (Table 2). We found that compared to control sites, pH levels were 1.1× higher under both winter nests (*p* = 0.037) and burrows (*p* = 0.043).

#### 3.2.4 Conductivity

We observed a structure × habitat interaction for soil conductivity (Table 2). We found differences in soil conductivity between habitats and structure types, but not between structure types and control sites within either habitat type (*p* > 0.10, Figure S8).

#### 3.2.5 Soil Respiration

Though we observed a structure × habitat interaction for soil respiration (Table 2), there were no significant effects detected during pairwise analyses.

## 4 Discussion

Our results reiterate the importance of small mammal structures in influencing biogeochemical cycling in the Arctic. We found that different lemming structures had varying and influential potential bottom‐up regulation of tundra ecosystems through effects on both below‐ and above‐ground processes and properties. Not only did structures affect nutrient pools in soils, such as decreases in C, N, and P pools under burrows, but we also showed influences on above‐ground variables such as vegetation diversity. It is probable that the creation of structures affected plant communities (e.g., clearing vegetation to maintain runways), which then also influences below‐ground processes and nutrient availability, feeding back into unique plant communities at different structure types. Due to the uneven spatial distribution of structure types (Roy et al. 2022) and their effects within habitat types, lemming structures may help create or maintain spatial heterogeneity of biogeochemical cycling in tundra systems and may be important in regulating ecosystem responses to change by increasing productivity and nutrient cycling rates (García‐Palacios et al. 2011) during the peak of their population cycle (Roy et al. 2022). Finally, we showed that the effects of these structures vary between contemporary (flat‐centered polygon) and potential future (high‐centered polygon) habitat types and may have implications for the future of ecosystem function in changing arctic systems.

We observed lemming structures to have influences on plant community composition. It was interesting that we observed certain plant species and plant groups at particular structure types (Figure 3, Table S1). Roy (2022, unpublished data) did not find similar effects on species diversity or occurrence between structure types in similar flat‐centered polygonal tundra, but they had used larger quadrats (40 × 40 cm). It is possible that the effects we observed may have been influenced by our relatively small sampling scale, sample size, or the fact that species may have been obscured by winter nests during sampling by over‐ or underrepresenting certain plant groups or species in relation to structures. Additionally, some structures (e.g., winter nests) may have obscured some plant species and biased detection ability in cover estimates. Finally, it is possible that some effects would not have been detectable on a different sampling scale (e.g., 1 m^2^). We recommend that future research determine what the area of impact is surrounding structures, both in terms of above‐ and below‐ground variables. Despite these uncertainties, similar effects of structures on plant communities have been seen with other animal structures in tundra and alpine systems: plateau pika ( *Ochotona curzoniae* ) and Stoliczka's vole ( *Alticola stoliczkanus* , Bagchi et al. 2006); Tatra marmot ( *Marmota marmota latirostris* , Ballová et al. 2019); Arctic fox ( *Vulpes lagopus* , Fafard et al. 2020). Additionally, our results may have been influenced though lemmings' potential selection of certain microhabitats (vegetation communities, soils) to construct structures, but the construction and maintenance of these structures likely have additive effects on the vegetation and habitat. As our control locations were in the same habitat as structure sites, this should have accounted for any potential habitat selection by lemmings. Furthermore, during peak abundance years, structures are very prevalent (McKendrick et al. 1980; Roy et al. 2022) and there is no apparent habitat preference within habitat types (Roy, personal observation). The effects of structures on plant community composition were potentially caused by selective foraging or consumption (Batzli and Pitelka 1983), use of certain species in structure building (e.g., sedges mainly used in winter nests, Roy et al. 2022), or changes in habitat conditions (e.g., nutrient availability, Fafard et al. 2020; water availability, Laundre 1993; light availability, Borer et al. 2014) which could support these species. The effects we observed of brown lemming structures on plant communities and multiple nutrient pools may be localized, but can have impacts over larger spatial areas. As lemming structures can cover a significant portion of their habitats during periods of high lemming abundance (up to 12.5% of the landscape, Roy et al. 2022) and the impacts of structures on nutrients may extend up to 2 m from the structure location in some systems (Ross et al. 2007), structures may aid in regulating broader spatial areas than their individual footprints may imply. Minor changes in plant communities, especially at larger scales, might aid in maintaining heterogeneity of species composition and above‐ground processes in tundra systems. This may be especially impactful in ecosystems like the Arctic, where vegetation turnover is relatively slow (Callaghan and Jonasson 1995). Though less abundant than winter nests (Roy et al. 2022), the effects we observed of burrows and latrines may result in higher amounts of bare ground and lower litter cover, which would still alter ecosystem processes at local scales (Fafard et al. 2020) with potential impacts on landscape‐level productivity and C‐storage (Bret‐Harte et al. 2008; Johnson et al. 2011). Changes in plant community assemblage and the amount of vegetation cover caused by small mammal structures, particularly in years of high abundance, may have regulatory influence on ecosystem function in this system.

**FIGURE 8:**
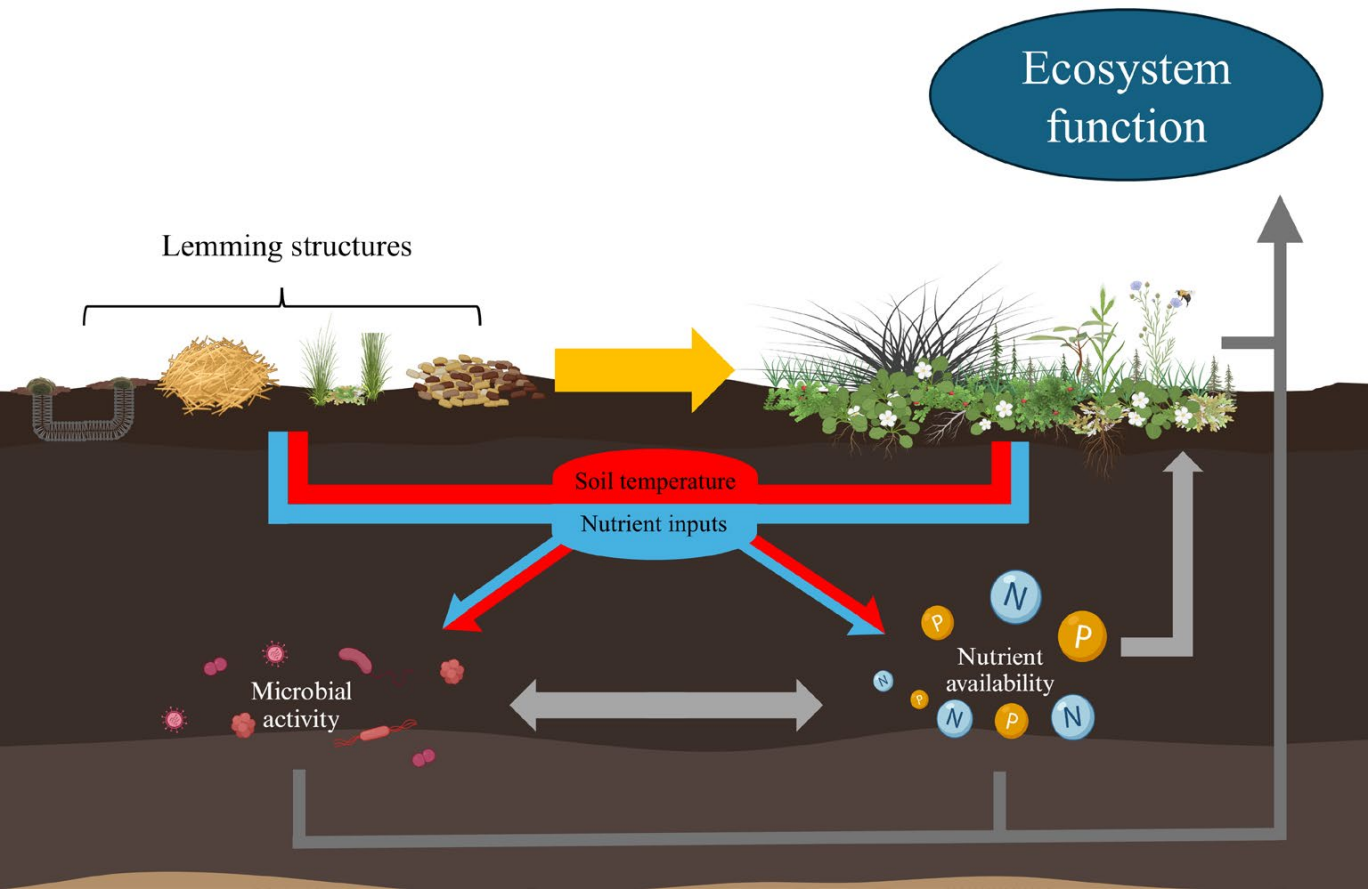
Conceptual diagram depicting direct and indirect interactions and effects of Brown lemming ( *Lemmus trimucronatus* ) structures on ecosystem function in arctic polygonal tundra.

Similar to above ground processes, we also observed the effect of structures on below‐ground factors, with these effects having the potential to influence local and broader tundra ecosystem function. Roy et al. (2022) found that structures, in particular small mammal winter nests (i.e., hay piles), affected below‐ground N pools and could alter the strength of nutrient limitation and influence the ability of the tundra systems to act as a C sink or source. Compared to this study in the same system (Roy et al. 2022), we observed some similar effects of soils under winter nests having increased N pools and soils under latrines having increased P and C pools, but we observed overall fewer effects on nutrient pools of winter nests than seen previously. Presently, we sampled burrows for the first time and show that they have an influence on shaping below‐ground biogeochemical processes by having more numerous and stronger effects on nutrient pools than other structure types, tending to have lower soil nutrient availability and exo‐enzyme activities than sites without lemming structures (controls) in both habitat types (Figure 6). Given the relatively strong (1.2–7.5×) decrease in C concentrations and C‐acquiring enzyme activities at burrows compared to controls (Figure 6), burrows may act as routes for C loss on the tundra. In addition to maintaining heterogeneity of above‐ground processes, burrows may be important to maintaining spatial heterogeneity of below‐ground processes across tundra habitats (Egelkraut et al. 2018; Fafard et al. 2020). It is of note that the impacts of structures on soils observed here varied in terms of structure type–nutrient type relationship and effect size compared to previous studies in the same system. Roy et al. (2022) found relatively strong impacts of winter nests on multiple soil N and P pools, but we found that winter nests affected relatively fewer nutrient pools (only ${\mathrm{NH}}_{4}^{+}$). The reason for this disparity in results is unclear; however, we suspect that the differences in effects may have been due to one or a combination of various factors: (1) differences in sample timing and the strong variation in seasonal availability of soil nutrients in arctic systems (McLaren et al. 2018), (2) that previously unsampled burrows had stronger effects than other structure types, or (3) that we sampled an additional habitat type compared to Roy et al. (2022). Though not examined here, these differences in soil nutrients between structure types can feedback to affect plant nutrient content (Tuomi et al. 2019; Petit Bon et al. 2020; Roy et al. 2022) and influence associated ecosystem processes within each tundra type uniquely. Despite differences in specific nutrient or structure types, the data presented here reaffirm the influence of these small mammal structures in regulating local nutrient cycling with potential feedbacks on landscape processes (Olofsson et al. 2012; Lara et al. 2017; Koltz et al. 2022; Figure 8).

We also begin to elucidate the potential mechanisms through which structures affect biogeochemical processes. The removal of litter and vegetation cover via trail grooming and trampling at latrines and burrows, resulting in higher bare ground cover, seems to be a driver of the effects of these structure types. Though not seen here, increases in the amount of bare ground are directly associated with soil temperatures, and because of bare ground's influence on soil temperatures and nutrient cycling rates (Stark 2007), these temperature differences help to explain some of the observed lower nutrient concentrations and exo‐enzyme activity rates at latrine and burrow sites (Figure 6). The increases in soil N and P we observed at latrines were possibly due to nutrient inputs from urine and feces (Sitters and Olde Venterink 2021); although, potentially also partially due to changes in vegetation and litter cover. In addition to differences in bare ground, the structure of the vegetation community may also be a factor regulating the effects of each structure type. With different structure types promoting unique plant species and assemblages of functional groups, they can help to regulate both the quantity and quality (species identity) of litter (Tuomi et al. 2019). These alterations in litter quantity and quality have influences on other biogeochemical drivers such as soil temperature, pH, and conductivity (as seen here), in addition to other factors, which interact to affect biogeochemical cycling through increasing or decreasing nutrient availability and cycling rates (Gu et al. 1994; Whitford and Steinberger 2010; Zheng et al. 2018). The insulative litter from winter nests themselves was a likely driver for the lower soil temperatures observed at nest sites (Figure 7), and helped maintain temperature conditions similar to controls, leading to the fewer below‐ground effects we observed at these sites. Alternatively, the accumulation of decomposing *Carex aquatilis* , a relatively high N‐quality litter species (Shaver et al. 1979), at nest sites (Roy et al. 2022) ultimately led to increases in soil N. Further work is needed to gain a more detailed understanding of each potential mechanism and their individual importance in regulating ecosystem processes. By understanding how these structures influence biogeochemical processes, we can begin to examine how their effects may be altered under changing environmental conditions.

Perhaps our most interesting finding is that the effects of structures on biogeochemical variables differed between habitat types. It was likely that the effects we observed were due to interactions between lemming activities and state factors (e.g., soil moisture, pH) within each habitat type. We saw that burrows had stronger effects and affected more below‐ground variables (Total C and N, ${\mathrm{NH}}_{4}^{+}$, ${\mathrm{PO}}_{4}^{3-}$, EOC, ETN, MBC, and MBN) in flat‐centered polygon (contemporary) tundra and that latrines affected more below‐ground variables (${\mathrm{PO}}_{4}^{3-}$, EOC) in high‐centered polygon (potential future) tundra conditions. Additionally, if high‐centered polygon tundra is representative of future tundra conditions, then the potential effects in high‐centered polygon compared to flat‐centered polygon tundra of latrines (higher C and P availability) and burrows (stronger impacts of enzyme activity but lesser impacts on total and available nutrients, Figure 6) have implications for tundra ecosystem function through potential regulation of primary productivity and decomposition (Wardle et al. 2002; Tuomi et al. 2019), influencing how the ecosystem acts as a C sink or source into the future (Johnson et al. 2011; Min et al. 2021). The differences we observed in effect type and size between habitat types suggest that as high‐centered polygonal tundra becomes more prevalent, brown lemmings use of these altered habitats due to climate change may help regulate tundra biogeochemical processes in these transformed landscapes. Though this study was focused on local effects of ecosystem processes within one tundra system in Northern Alaska, many characteristics of this system are shared among other polygonal tundra systems across the Arctic. Future studies should examine whether the effects we observed here are ubiquitous across these polygonal tundra systems and other tundra ecosystem types undergoing change. Furthermore, if future arctic conditions alter the density of lemmings within their habitats for extended periods of time, we may expect effects of these herbivores on the ecosystem to also be modified (Koltz et al. 2022) through changes in the abundance or density of their structures. Changes in herbivore community structure under climate change may also alter the effects of structure builders in this system (Barbero‐Palacios et al. 2024). As collared lemmings may use future conditions (high‐centered polygon tundra) differently than brown lemmings, it was unfortunate that we were unable to sample at collared lemming structure sites because of low collared lemming numbers in the year we sampled. There are differences between the two species as they consume different forage and potentially use habitats in unique ways (Batzli and Pitelka 1983); however, as brown lemmings generally reach higher densities than collared lemmings (Gruyer et al. 2008) it is probable that they would have stronger effects than those of collared lemmings due to differences in species' abundances. Alternatively, it is possible that lemmings will be replaced altogether as the dominant small mammal herbivore in the future (e.g., vole [*Microtus* spp.] expansion north, Sokolova et al. 2024). Though the effects of lemming and vole structures are relatively similar within their respective habitats (Roy et al. 2022), the species' ecologies and effects of their structures do vary and it is unclear how the structures of a completely novel species will interact with changing arctic systems. As arctic tundra ecosystems are rapidly changing (e.g., vegetation communities, herbivore communities, topography, climate), research needs to include multiple ecological scenarios to best predict the influence of herbivores and their structures on ecosystem function in the future.

In this study we elucidate how the impacts of small mammal structures alter ecosystem processes within two tundra types that represent contemporary and future tundra conditions. We found that structures built by brown lemmings affect both below‐ground variables and also plant communities to some extent, with implications for primary production and decomposition in this nutrient‐limited system. The effects we observed most likely will vary depending on future conditions across the Arctic; as such we recommend further studies to incorporate multiple regions and tundra types across where herbivore composition and activity may potentially change (Sokolova et al. 2024). Although the effects of lemming structures appear to vary between years (Roy et al. 2022), here we provide continued evidence of the importance of small mammals in affecting arctic ecosystem function through regulation of biogeochemical processes, both as structure builders and herbivores (Johnson et al. 2011; Roy et al. 2020, 2022; Min et al. 2021). By creating structures and altering portions of their habitats, small mammal herbivores have some regulatory control over ecosystem function. The findings and data we provide can be used to update ecological models (e.g., Rastetter et al. 2022) and better predict the future of arctic ecosystem function under continuing environmental changes.

## Author Contributions

**Austin Roy:** conceptualization (equal), data curation (equal), formal analysis (equal), funding acquisition (equal), investigation (equal), methodology (equal), project administration (equal), writing – original draft (lead). **Jennie R. McLaren:** conceptualization (equal), funding acquisition (lead), investigation (equal), project administration (equal), resources (lead), supervision (lead), writing – review and editing (equal).

## Conflicts of Interest

The authors declare no conflicts of interest.

## Supporting information


Data S1.

## Data Availability

Data from this project is available through the Arctic Data Center: Roy and McLaren 2023, Soil and plant variables collected at brown lemming structures near Utqiaġvik, Alaska, summer 2021 (https://doi.org/10.18739/A2GX44W4K).

## References

[ece371523-bib-0001] Abolt, C. J. , M. H. Young , A. L. Atchley , and C. J. Wilson . 2019. “Brief Communication: Rapid Machine‐Learning‐Based Extraction and Measurement of Ice Wedge Polygons in High‐Resolution Digital Elevation Models.” Cryosphere 13: 237–245.

[ece371523-bib-0002] Assmann, J. J. , I. H. Myers‐Smith , A. B. Phillimore , et al. 2019. “Local Snow Melt and Temperature‐But Not Regional Sea Ice‐Explain Variation in Spring Phenology in Coastal Arctic Tundra.” Global Change Biology 25: 2258–2274. 30963662 10.1111/gcb.14639

[ece371523-bib-0003] Bagchi, S. , T. Namgail , and M. E. Ritchie . 2006. “Small Mammalian Herbivores as Mediators of Plant Community Dynamics in the High‐Altitude Arid Rangelands of Trans‐Himalaya.” Biological Conservation 127: 438–442.

[ece371523-bib-0004] Ballová, Z. , L. Pekárik , V. Píš , and J. Šibík . 2019. “How Much Do Ecosystem Engineers Contribute to Landscape Evolution? A Case Study on Tatra Marmots.” Catena 182: 104121.

[ece371523-bib-0005] Baltensperger, A. P. , and F. Huettmann . 2015. “Predicted Shifts in Small Mammal Distributions and Biodiversity in the Altered Future Environment of Alaska: An Open Access Data and Machine Learning Perspective.” PLoS One 10: e0132054. 26207828 10.1371/journal.pone.0132054 PMC4514745

[ece371523-bib-0006] Barbero‐Palacios, L. , I. C. Barrio , M. García Criado , et al. 2024. “Herbivore Diversity Effects on Arctic Tundra Ecosystems: A Systematic Review.” Environmental Evidence 13: 1–21. 10.1186/s13750-024-00330-9. 39294685 PMC11378771

[ece371523-bib-0007] Barton, K. 2022. “MuMIn: Multi‐Model Inference.” https://CRAN.R‐project.org/package=MuMIn430B.

[ece371523-bib-0008] Bates, D. , M. Mächler , B. Bolker , and S. Walker . 2015. “Fitting Linear Mixed‐Effects Models Using lme4.” Journal of Statistical Software 67: 1–48.

[ece371523-bib-0010] Batzli, G. O. , and F. A. Pitelka . 1983. “Nutritional Ecology of Microtine Rodents: Food Habits of Lemmings Near Barrow, Alaska.” Journal of Mammalogy 64: 648–655.

[ece371523-bib-0011] Batzli, G. O. , F. A. Pitelka , and G. N. Cameron . 1983. “Habitat Use by Lemmings Near Barrow, Alaska.” Holarctic Ecology 6: 255–262.

[ece371523-bib-0012] Batzli, G. O. , R. G. White , S. F. MacLean , F. A. Pitelka , and B. D. Collier . 1980. “The Herbivore‐Based Trophic System.” In An Arctic Ecosystem: The Coastal Tundra at Barrow, Alaska, edited by J. Brown , P. C. Miller , L. Tieszen , and F. L. Bunnell , 335–410. Dowden, Hutchinson & Ross Inc.

[ece371523-bib-0079] Beylich, A. , H.‐R. Oberholzer , S. Schrader , H. Höper , and B.‐M. Wilke . 2010. “Evaluation of Soil Compaction Effects on Soil Biota and Soil Biological Processes in Soils.” Soil and Tillage Research 109: 133–143.

[ece371523-bib-0013] Borer, E. T. , E. W. Seabloom , D. S. Gruner , et al. 2014. “Herbivores and Nutrients Control Grassland Plant Diversity via Light Limitation.” Nature 508: 517–520. 24670649 10.1038/nature13144

[ece371523-bib-0014] Bret‐Harte, M. S. , M. C. Mack , G. R. Goldsmith , et al. 2008. “Plant Functional Types Do Not Predict Biomass Responses to Removal and Fertilization in Alaskan Tussock Tundra.” Journal of Ecology 96: 713–726. 18784797 10.1111/j.1365-2745.2008.01378.x PMC2438444

[ece371523-bib-0015] Britton, M. E. 1957. “Vegetation of the Arctic Tundra.” In Arctic Biology, edited by H. P. Hansen , 26–72. Oregon State Univ. Press.

[ece371523-bib-0016] Brookes, P. C. , A. Landman , G. Pruden , and D. S. Jenkinson . 1985. “Chloroform Fumigation and the Release of Soil Nitrogen: A Rapid Direct Extraction Method to Measure Microbial Biomass Nitrogen in Soil.” Soil Biology and Biochemistry 17: 837–842.

[ece371523-bib-0017] Callaghan, T. V. , and S. Jonasson . 1995. “Arctic Terrestrial Ecosystems and Environmental Change.” Philosophical Transactions of the Royal Society of London. Series A: Physical and Engineering Sciences 352: 259–276.

[ece371523-bib-0018] D'Angelo, E. , J. Crutchfield , and M. Vandiviere . 2001. “Rapid, Sensitive, Microscale Determination of Phosphate in Water and Soil.” Journal of Environmental Quality 30: 2206–2209. 11790034 10.2134/jeq2001.2206

[ece371523-bib-0019] Doane, T. A. , and W. R. Horwath . 2003. “Spectrophotometric Determination of Nitrate With a Single Reagent.” Analytical Letters 36: 2713–2722.

[ece371523-bib-0020] Egelkraut, D. , K.‐Å. Aronsson , A. Allard , M. Åkerholm , S. Stark , and J. Olofsson . 2018. “Multiple Feedbacks Contribute to a Centennial Legacy of Reindeer on Tundra Vegetation.” Ecosystems 21: 1545–1563.

[ece371523-bib-0021] Egelkraut, D. , H. Barthelemy , and J. Olofsson . 2020. “Reindeer Trampling Promotes Vegetation Changes in Tundra Heathlands: Results From a Simulation Experiment.” Journal of Vegetation Science 31: 476–486.

[ece371523-bib-0022] Fafard, P. M. , J. D. Roth , and J. H. Markham . 2020. “Nutrient Deposition on Arctic Fox Dens Creates Atypical Tundra Plant Assemblages at the Edge of the Arctic.” Journal of Vegetation Science 31: 172–179.

[ece371523-bib-0023] Fox, J. , and S. Weisberg . 2019. An {R} Companion to Applied Regression. 2nd ed. Sage Publications.

[ece371523-bib-0024] French, H. M. 2007. The Periglacial Environment. 3rd ed. John Wiley and Sons.

[ece371523-bib-0025] García‐Palacios, P. , F. T. Maestre , and A. Gallardo . 2011. “Soil Nutrient Heterogeneity Modulates Ecosystem Responses to Changes in the Identity and Richness of Plant Functional Groups.” Journal of Ecology 99: 551–562. 25914424 10.1111/j.1365-2745.2010.01765.x PMC4407982

[ece371523-bib-0026] Gough, L. , and D. R. Johnson . 2018. “Mammalian Herbivory Exacerbates Plant Community Responses to Long‐Term Increased Soil Nutrients in Two Alaskan Tundra Plant Communities.” Arctic Science 4: 153–166.

[ece371523-bib-0027] Gruyer, N. , G. Gauthier , and D. Berteaux . 2008. “Cyclic Dynamics of Sympatric Lemming Populations on Bylot Island, Nunavut, Canada.” Canadian Journal of Zoology 86: 910–917.

[ece371523-bib-0028] Gu, B. , J. Schmitt , Z. Chen , L. Liang , and J. F. McCarthy . 1994. “Adsorption and Desorption of Natural Organic Matter on Iron Oxide: Mechanisms and Models.” Environmental Science & Technology 28: 38–46. 22175831 10.1021/es00050a007

[ece371523-bib-0029] Harrington, R. , I. Woiwod , and T. Sparks . 1999. “Climate Change and Trophic Interactions.” Trends in Ecology & Evolution 14: 146–150. 10322520 10.1016/s0169-5347(99)01604-3

[ece371523-bib-0030] Hinkel, K. M. , R. C. Frohn , F. E. Nelson , W. R. Eisner , and R. A. Beck . 2005. “Morphometric and Spatial Analysis of Thaw Lakes and Drained Thaw Lake Basins in the Western Arctic Coastal Plain, Alaska.” Permafrost and Periglacial Processes 16: 327–341. 10.1002/ppp.532.

[ece371523-bib-0031] Hobbs, N. T. , and K. R. Searle . 2005. “A Reanalysis of the Body Mass Scaling of Trampling by Large Herbivores.” Oecologia 145: 462–464. 16001222 10.1007/s00442-005-0149-6

[ece371523-bib-0032] Hodkinson, I. D. , N. R. Webb , J. S. Bale , and W. Block . 1999. “Hydrology, Water Availability and Tundra Ecosystem Function in a Changing Climate: The Need for a Closer Integration of Ideas?” Global Change Biology 5: 359–369.

[ece371523-bib-0033] Johnson, D. R. , M. J. Lara , G. R. Shaver , G. O. Batzli , J. D. Shaw , and C. E. Tweedie . 2011. “Exclusion of Brown Lemmings Reduces Vascular Plant Cover and Biomass in Arctic Coastal Tundra: Resampling of a 50+ Year Herbivore Exclosure Experiment Near Barrow, Alaska.” Environmental Research Letters 6: 045507.

[ece371523-bib-0034] Koltz, A. M. , L. Gough , and J. R. McLaren . 2022. “Herbivores in Arctic Ecosystems: Effects of Climate Change and Implications for Carbon and Nutrient Cycling.” Annals of the New York Academy of Sciences 1516: 28–47. 35881516 10.1111/nyas.14863 PMC9796801

[ece371523-bib-0035] Lara, M. J. , D. R. Johnson , C. Andresen , R. D. Hollister , and C. E. Tweedie . 2017. “Peak Season Carbon Exchange Shifts From a Sink to a Source Following 50+ Years of Herbivore Exclusion in an Arctic Tundra Ecosystem.” Journal of Ecology 105: 122–131.

[ece371523-bib-0036] Lara, M. J. , A. D. McGuire , E. S. Euskirchen , et al. 2015. “Polygonal Tundra Geomorphological Change in Response to Warming Alters Future CO_2_ and CH_4_ Flux on the Barrow Peninsula.” Global Change Biology 21: 1634–1651. 25258295 10.1111/gcb.12757

[ece371523-bib-0037] Laundre, J. W. 1993. “Effects of Small Mammal Burrows on Water Infiltration in a Cool Desert Environment.” Oecologia 94: 43–48. 28313856 10.1007/BF00317299

[ece371523-bib-0038] Lenth, R. V. , B. Bolker , P. Buerkner , et al. 2023. “emmeans: Estimated Marginal Means, aka Least‐Squares Means.” Accessed December 18, 2023. https://cran.r‐project.org/web/packages/emmeans/index.html.

[ece371523-bib-0039] Liljedahl, A. K. , J. Boike , R. P. Daanen , et al. 2012. “Ice‐Wedge Polygon Type Controls Low‐Gradient Watershed‐Scale Hydrology.” In Proceedings of the Tenth International Conference on Permafrost. Northern Publisher.

[ece371523-bib-0040] Liljedahl, A. K. , J. Boike , R. P. Daanen , et al. 2016. “Pan‐Arctic Ice‐Wedge Degradation in Warming Permafrost and Its Influence on Tundra Hydrology.” Nature Geoscience 9: 312–318.

[ece371523-bib-0041] McKendrick, J. D. , G. O. Batzli , K. R. Everett , and J. C. Swanson . 1980. “Some Effects of Mammalian Herbivores and Fertilization on Tundra Soils and Vegetation.” Arctic and Alpine Research 12: 565–578.

[ece371523-bib-0042] McLaren, J. R. , K. M. Buckeridge , M. J. Van de Weg , G. R. Shaver , J. P. Schimel , and L. Gough . 2017. “Shrub Encroachment in Arctic Tundra: *Betula nana* Effects on Above‐ and Belowground Litter Decomposition.” Ecology 98: 1361–1376. 28263375 10.1002/ecy.1790

[ece371523-bib-0043] McLaren, J. R. , A. Darrouzet‐Nardi , M. N. Weintraub , and L. Gough . 2018. “Seasonal Patterns of Soil Nitrogen Availability in Moist Acidic Tundra.” Arctic Science 4: 98–109.

[ece371523-bib-0044] Min, E. , S. Naeem , L. Gough , et al. 2023. “Using Structure to Model Function: Incorporating Canopy Structure Improves Estimates of Ecosystem Carbon Flux in Arctic Dry Heath Tundra.” Environmental Research Letters 18: 065004.

[ece371523-bib-0045] Min, E. , M. E. Wilcots , S. Naeem , et al. 2021. “Herbivore Absence Can Shift Dry Heath Tundra From Carbon Source to Sink During Peak Growing Season.” Environmental Research Letters 16: 024027.

[ece371523-bib-0046] Morris, D. W. , D. L. Davidson , and C. J. Krebs . 2000. “Measuring the Ghost of Competition: Insights From Density‐Dependent Habitat Selection on the Co‐Existence and Dynamics of Lemmings.” Evolutionary Ecology Research 2: 41–67.

[ece371523-bib-0047] Morris, D. W. , and A. Dupuch . 2012. “Habitat Change and the Scale of Habitat Selection: Shifting Gradients Used by Coexisting Arctic Rodents.” Oikos 121: 975–984.

[ece371523-bib-0048] Nitzbon, J. , M. Langer , S. Westermann , L. Martin , K. Schanke Aas , and J. Boike . 2019. “Pathways of Ice‐Wedge Degradation in Polygonal Tundra Under Different Hydrological Conditions.” Cryosphere 13: 1089–1123.

[ece371523-bib-0049] Nyland, K. E. , A. E. Klene , J. Brown , et al. 2017. “Traditional Iñupiat Ice Cellars (SIĠḷUAQ) in Barrow, Alaska: Characteristics, Temperature Monitoring, and Distribution.” Geographical Review 107: 143–158.

[ece371523-bib-0050] Oksanen, J. , G. Simpson , F. Blanchet , et al. 2022. “vegan: Community Ecology Package.” R Package Version 2:6‐4. https://CRAN.R‐project.org/package=vegan.

[ece371523-bib-0051] Olefeldt, D. , S. Goswami , G. Grosse , et al. 2016. “Circumpolar Distribution and Carbon Storage of Thermokarst Landscapes.” Nature Communications 7: 13043. 10.1038/ncomms13043. PMC5062615 27725633

[ece371523-bib-0052] Olofsson, J. , H. Tommervik , and T. V. Callaghan . 2012. “Vole and Lemming Activity Observed From Space.” Nature Climate Change 2: 880–883.

[ece371523-bib-0053] Petit Bon, M. , K. Gunnarsdotter Inga , I. S. Jónsdóttir , T. A. Utsi , E. M. Soininen , and K. A. Bråthen . 2020. “Interactions Between Winter and Summer Herbivory Affect Spatial and Temporal Plant Nutrient Dynamics in Tundra Grassland Communities.” Oikos 129: 1229–1242.

[ece371523-bib-0080] R Core Team . 2023. “R: A Language and Environment for Statistical Computing.” R Foundation for Statistical Computing, Vienna, Austria.

[ece371523-bib-0054] Rastetter, E. B. , K. L. Griffin , R. J. Rowe , L. Gough , J. R. McLaren , and N. T. Boelman . 2022. “Model Responses to CO_2_ and Warming Are Underestimated Without Explicit Representation of Arctic Small‐Mammal Grazing.” Ecological Applications 32: e02478. 34657358 10.1002/eap.2478 PMC9285540

[ece371523-bib-0055] Rhine, E. D. , R. L. Mulvaney , E. J. Pratt , and G. K. Sims . 1998. “Improving the Berthelot Reaction for Determining Ammonium in Soil Extracts and Water.” Soil Science Society of America Journal 62: 473–480.

[ece371523-bib-0056] Ross, B. E. , A. W. Reed , R. L. Rehmeier , G. A. Kaufman , and K. W. Kaufman . 2007. “Effects of Prairie Vole Runways on Tallgrass Prairie. 2007.” Transactions of the Kansas Academy of Science 110: 100–106.

[ece371523-bib-0057] Roy, A. 2022. “Understanding the Role of Small Mammals in Arctic Biogeochemical Cycling.” Dissertation, The University of Texas at El Paso.

[ece371523-bib-0058] Roy, A. , L. Gough , N. T. Boelman , R. J. Rowe , K. L. Griffin , and J. R. McLaren . 2022. “Small but Mighty: Impacts of Rodent‐Herbivore Structures on Carbon and Nutrient Cycling in Arctic Tundra.” Functional Ecology 36: 2331–2343.

[ece371523-bib-0059] Roy, A. , M. Suchocki , L. Gough , and J. R. McLaren . 2020. “Above‐ and Belowground Responses to Long‐Term Herbivore Exclusion.” Arctic, Antarctic, and Alpine Research 52: 109–119.

[ece371523-bib-0060] Saiya‐Cork, K. R. , R. L. Sinsabaugh , and D. R. Zak . 2002. “The Effects of Long Term Nitrogen Deposition on Extracellular Enzyme Activity in an *Acer saccharum* Forest Soil.” Soil Biology and Biochemistry 34: 1309–1315.

[ece371523-bib-0061] Selwood, K. E. , M. A. McGeoch , and R. Mac Nally . 2015. “The Effects of Climate Change and Land‐Use Change on Demographic Rates and Population Viability.” Biological Reviews of the Cambridge Philosophical Society 90: 837–853. 25155196 10.1111/brv.12136

[ece371523-bib-0062] Sharma, S. , S. Couturier , and S. D. Côté . 2009. “Impacts of Climate Change on the Seasonal Distribution of Migratory Caribou.” Global Change Biology 15: 2549–2562.

[ece371523-bib-0063] Shaver, G. R. , F. S. Chapin III , and W. D. Billings . 1979. “Ecotypic Variation in *Carex aquatilis* on Ice‐Wedge Polygons in the Alaska Coastal Tundra.” Journal of Ecology 67: 1025–1045.

[ece371523-bib-0064] Sistla, S. A. , J. C. Moore , R. T. Simpson , L. Gough , G. R. Shaver , and J. P. Schimel . 2013. “Long‐Term Warming Restructures Arctic Tundra Without Changing Net Soil Carbon Storage.” Nature 497: 615–618. 23676669 10.1038/nature12129

[ece371523-bib-0065] Sitters, J. , and H. Olde Venterink . 2021. “Stoichiometric Impact of Herbivore Dung Versus Urine on Soils and Plants.” Plant and Soil 462: 59–65.

[ece371523-bib-0066] Sokolova, N. A. , I. A. Fufachev , D. Ehrich , V. G. Shtro , V. A. Sokolov , and A. A. Sokolov . 2024. “Expansion of Voles and Retraction of Lemmings Over 60 Years Along a Latitudinal Gradient on Yamal Peninsula.” Global Change Biology 30: e17161.

[ece371523-bib-0067] Stark, S. 2007. “Nutrient Cycling in the Tundra.” In Nutrient Cycling in Terrestrial Ecosystems, 309–331. Springer.

[ece371523-bib-0068] Stark, S. , M. K. Männistö , and A. Eskelinen . 2015. “When Do Grazers Accelerate or Decelerate Soil Carbon and Nitrogen Cycling in Tundra? A Test of Theory on Grazing Effects in Fertile and Infertile Habitats.” Oikos 124: 593–602.

[ece371523-bib-0069] Streletskiy, D. A. , S. Clemens , J.‐P. Lanckman , and N. I. Shiklomanov . 2023. “The Costs of Arctic Infrastructure Damages due to Permafrost Degradation.” Environmental Research Letters 18: 015006.

[ece371523-bib-0070] Tuomi, M. , S. Stark , K. S. Hoset , et al. 2019. “Herbivore Effects on Ecosystem Process Rates in a Low‐Productive System.” Ecosystems 22: 827–843.

[ece371523-bib-0071] Van der Wal, R. , S. M. J. van Lieshout , and M. J. J. E. Loonen . 2001. “Herbivore Impact on Moss Depth, Soil Temperature and Arctic Plant Growth.” Polar Biology 24: 29–32.

[ece371523-bib-0072] Voroney, R. P. , P. C. Brooks , and R. P. Beyaert . 2006. “Soil Microbial Biomass C, N, P, and S.” In Soil Sampling and Methods of Analysis, edited by M. R. Carter and E. G. Gregorich . Lewis.

[ece371523-bib-0073] Wardle, D. A. , K. I. Bonner , and G. M. Barker . 2002. “Linkages Between Plant Litter Decomposition, Litter Quality, and Vegetation Responses to Herbivores.” Functional Ecology 16: 585–595.

[ece371523-bib-0074] Webber, P. J. 1978. “Spatial and Temporal Variation of the Vegetation and Its Productivity.” In Vegetation and Production Ecology of the Alaskan Arctic Tundra, edited by L. Tieszen , 37–112. Springer.

[ece371523-bib-0075] Webber, P. J. , P. C. Miller , F. S. Chapin , and B. H. McCown . 1980. “The Vegetation: Pattern and Succession.” In An Arctic Ecosystem: The Coastal Tundra at Barrow, Alaska, edited by J. Brown , P. C. Miller , L. Tieszen , and F. L. Bunnell , 186–218. Dowden, Hutchinson & Ross Inc.

[ece371523-bib-0076] Whitford, W. G. , and Y. Steinberger . 2010. “Pack Rats (Neotoma spp.): Keystone Ecological Engineers?” Journal of Arid Environments 74: 1450–1455.

[ece371523-bib-0077] Wrona, F. J. , M. Johansson , J. M. Culp , et al. 2016. “Transitions in Arctic Ecosystems: Ecological Implications of a Changing Hydrological Regime.” Journal of Geophysical Research: Biogeosciences 121: 650–674.

[ece371523-bib-0078] Zheng, J. , T. RoyChowdhury , Z. Yang , B. Gu , S. D. Wullschleger , and D. E. Graham . 2018. “Impacts of Temperature and Soil Characteristics on Methane Production and Oxidation in Arctic Tundra.” Biogeosciences 15: 6621–6635.

