# Supplementary material for: The Effect of Structure Building Small Mammals in a Shifting Arctic Landscape
Source: Ecol Evol. 2025 Jun 4;15(6):e71523. doi: 10.1002/ece3.71523 (PMC12137189; doi:10.1002/ece3.71523)

Supplemental Table 1. Mean relative percent cover of vascular plant species detected at brown lemming structure (winter nest (WIN), runway (RUN), latrine (LAT), and burrow (BUR)) and control (CON) locations within flat-centered polygon (FCP) and high-centered polygon (HCP) tundra near Utqiaġvik, Alaska, USA, during the summer of 2021. Ten replicates of each structure type and 10 control sites were sampled within each habitat type during the summer of 2021, near Utqiaġvik, Alaska, USA.

|  | Habitat | | | | | | | | | | |
| --- | --- | --- | --- | --- | --- | --- | --- | --- | --- | --- | --- |
|  |  |  | FCP |  |  |  |  |  | HCP |  |  |
|  | CON | WIN | LAT | RUN | BUR |  | CON | WIN | LAT | RUN | BUR |
| *Alopecurus alpinus* | 8.473064 | 9.340659 | 0.952381 | 0.30303 | 0.71429 |  | 0.344828 | 0 | 0 | 0 | 0 |
| *Arctophila latifolia* | 2.5 | 0.714286 | 4.285714 | 5.031746 | 5.90405 |  | 0 | 0 | 0 | 0 | 1.649581 |
| *Carex aquatilis* | 26.64633 | 35.59524 | 39.26599 | 22.60269 | 14.5486 |  | 25.29861 | 57.46667 | 52.53806 | 50.45666 | 10.84369 |
| *Dupontia fisheri* | 0.434783 | 2.380952 | 2.404762 | 10.60882 | 9.29906 |  | 0 | 2.5 | 1.428571 | 0 | 0.30303 |
| *Eriophorum angustifolium* | 10.39662 | 13.99267 | 16.09091 | 16.14973 | 0.43478 |  | 12.10746 | 22.26667 | 12.6679 | 10.51801 | 6.22845 |
| *Eriophorum russeolum* | 0 | 1.538462 | 0 | 0.47619 | 0 |  | 9.605263 | 0 | 0.222222 | 0.25 | 0.121951 |
| *Eriophorum vaginatum* | 0 | 0 | 0 | 0 | 0.19231 |  | 0.526316 | 0 | 0 | 0 | 0 |
| *Luzula arctica* | 0 | 1.666667 | 6.795367 | 3.502825 | 1.30435 |  | 0 | 0 | 0 | 0 | 1.666667 |
| *Luzula confusa* | 0 | 0 | 0 | 0 | 0 |  | 0 | 0 | 0 | 0 | 1.081081 |
| *Oxyria digyna* | 5.333333 | 0 | 0 | 0 | 0 |  | 0 | 0 | 0 | 0 | 0 |
| *Pedicularis spp.* | 0 | 0 | 0 | 0 | 0 |  | 0 | 0 | 0 | 0 | 1.666667 |
| *Petasites frigidus* | 18.6728 | 11.79487 | 11.72829 | 10.44934 | 20.0488 |  | 37.57304 | 12.4 | 26.3523 | 24.82694 | 31.57358 |
| *Poa arctica* | 5.27108 | 1.25 | 6.327693 | 5.125354 | 13.4862 |  | 4.384181 | 0 | 0.465116 | 1.323338 | 15.85825 |
| *Polygonium* spp. | 0 | 0 | 0 | 0.909091 | 0 |  | 0 | 0 | 0 | 0 | 0 |
| *Potentilla hyparctica* | 0 | 0 | 0 | 0 | 0 |  | 0 | 0 | 0 | 0 | 0.27027 |
| *Rannuculus nivalis* | 0.851449 | 2.857143 | 0 | 0.909091 | 0.37037 |  | 1.022794 | 2.9 | 0 | 0.625 | 0 |
| *Salix pulchra* | 0 | 0 | 0.434783 | 1.782531 | 0 |  | 2.55814 | 0 | 0 | 0 | 14.9094 |
| *Salix rotundifolia* | 14.90909 | 5.14881 | 5.654674 | 12.84365 | 26.2405 |  | 0 | 0 | 0 | 0 | 0 |
| *Saxifraga cernua* | 0.37037 | 1.428571 | 1.201006 | 1.464646 | 0.62037 |  | 5.456979 | 2.066667 | 5.035021 | 10.21158 | 7.659976 |
| *Saxifraga foliolosa* | 0 | 0 | 0 | 0 | 0.38462 |  | 0 | 0 | 0 | 0 | 0 |
| *Saxifraga hieracifolia* | 0 | 0 | 0 | 0 | 0 |  | 0 | 0 | 0 | 0 | 0 |
| *Saxifraga punctata* | 4.847826 | 0.625 | 1.540541 | 0 | 1.30435 |  | 0 | 0 | 0.232558 | 0 | 4.864865 |
| *Stellaria laeta* | 0.709916 | 1.666667 | 0.761905 | 0.285714 | 0.25 |  | 0.318745 | 0.4 | 0.869565 | 1.788462 | 0.466349 |
| Unknown species 1 | 0 | 0 | 0.27027 | 5 | 0 |  | 0.136986 | 0 | 0 | 0 | 0 |
| Unknown species 2 | 0 | 0 | 0 | 0 | 0 |  | 0 | 0 | 0 | 0 | 0 |
| Unknown species 3 | 0 | 0 | 0 | 0 | 4.23077 |  | 0 | 0 | 0 | 0 | 0 |
| Unknown species 4 | 0.416667 | 0 | 0 | 0.555556 | 0 |  | 0 | 0 | 0 | 0 | 0 |
| Unknown species 5 | 0 | 0 | 0 | 0 | 0 |  | 0 | 0 | 0 | 0 | 0 |
| Unknown forb 1 | 0 | 0 | 2.285714 | 0 | 0.66667 |  | 0 | 0 | 0.188679 | 0 | 0.562219 |
| Unknown forb 2 | 0 | 0 | 0 | 0 | 0 |  | 0.666667 | 0 | 0 | 0 | 0.136986 |
| Unknown forb 3 | 0 | 0 | 0 | 0 | 0 |  | 0 | 0 | 0 | 0 | 0.136986 |
| Unknown forb 4 | 0 | 0 | 0 | 0 | 0 |  | 0 | 0 | 0 | 0 | 0 |
| Unknown forb 5 | 0 | 0 | 0 | 0 | 0 |  | 0 | 0 | 0 | 0 | 0 |

Supplemental Table 2. Pairwise comparison of soil enzyme activities at brown lemming structure sites (winter nest (WIN), runway (RUN), latrine (LAT), and burrow (BUR)) to control (CON) sites within flat-centered polygon (FCP) and high-centered polygon (HCP) tundra near Utqiaġvik, Alaska, USA. Bolded values represent statistically significant results (p < 0.05) and italicized values represent non-significant trends (0.05 < p < 0.10). Non-bolded p-values < 0.05 showed statistically non-significant results during post hoc tests. Ten replicates of each structure type and 10 control sites were sampled within each habitat type during the summer of 2021, near Utqiaġvik, Alaska, USA.

|  | FCP | | | |  | HCP | | | |
| --- | --- | --- | --- | --- | --- | --- | --- | --- | --- |
|  | WIN | LAT | RUN | BUR |  | WIN | LAT | RUN | BUR |
| β-glucosidase | 1.000 | **0.001** | 1.000 | **0.015** |  | 1.000 | 0.005 | 1.000 | **<0.001** |
| β-cellobiosidase | 1.000 | **<0.001** | 1.000 | *0.076* |  | 1.000 | *0.090* | 1.000 | **<0.001** |
| β-xylosidase | 1.000 | **<0.001** | 1.000 | 0.263 |  | 1.000 | **<0.001** | 0.502 | **<0.001** |
| α-glucosidase | 1.000 | **0.016** | 1.000 | 0.237 |  | 1.000 | 1.000 | 1.000 | **0.001** |
| LAP | 1.000 | 1.000 | 1.000 | 0.333 |  | 1.000 | 0.648 | 1.000 | 1.000 |
| NAG | 0.863 | **<0.001** | 0.379 | **<0.001** |  | 1.000 | **<0.001** | 0.467 | **<0.001** |
| Phosphatase | 1.000 | **<0.001** | 1.000 | **<0.001** |  | 1.000 | **<0.001** | 0.329 | **<0.001** |
| Phosphodiesterase | 1.000 | **<0.001** | 1.000 | *0.079* |  | 0.459 | 0.639 | 1.000 | **<0.001** |
| Phenol oxidase | - | - | - | - |  | - | - | - | - |
| Peroxidase | 1.000 | 1.000 | 1.000 | 1.000 |  | - | - | - | - |

List of Supplemental Figures

Supplemental Figure 1. Box plots showing differences, as determined through GLMMs, in mean % cover of live cover classes at brown lemming structures (HAY = hay pile, LAT = latrine, RUN = runway, BUR = burrow) and control site (CON) locations within flat-centered polygon (FCP) and high-centered polygon (HCP) tundra near Utqiaġvik, Alaska, USA. Ten replicates of each structure type and 10 control sites were sampled within each habitat type during the summer of 2021.

Supplemental Figure 2. Box plots showing differences, as determined through GLMMs, in mean % cover of litter and bare ground cover classes at brown lemming structures (HAY = hay pile, LAT = latrine, RUN = runway, BUR = burrow) and control site (CON) locations within flat-centered polygon (FCP) and high-centered polygon (HCP) tundra near Utqiaġvik, Alaska, USA. Ten replicates of each structure type and 10 control sites were sampled within each habitat type during the summer of 2021.

Supplemental Figure 3. Box plots showing differences, as determined through GLMMs, in a) total % carbon (% C), b) total % nitrogen (% N), and c) total % phosphorus (% P) at brown lemming structures (HAY = hay pile, LAT = latrine, RUN = runway, BUR = burrow) and control site (CON) locations within flat-centered polygon (FCP) and high-centered polygon (HCP) tundra near Utqiaġvik, Alaska, USA. Ten replicates of each structure type and 10 control sites were sampled within each habitat type during the summer of 2021.

Supplemental Figure 4. Box plots showing differences, as determined through GLMMs, in a) nitrate (NO_3_^-^), b) ammonium (NH_4_^+^), and c) phosphate (PO_4_^3-^) at brown lemming structures (HAY = hay pile, LAT = latrine, RUN = runway, BUR = burrow) and control site (CON) locations within flat-centered polygon (FCP) and high-centered polygon (HCP) tundra near Utqiaġvik, Alaska, USA. Ten replicates of each structure type and 10 control sites were sampled within each habitat type during the summer of 2021.

Supplemental Figure 5. Box plots showing differences, as determined through GLMMs, in a) extractable organic carbon (EOC) and b) extractable total nitrogen (ETN) at brown lemming structures (HAY = hay pile, LAT = latrine, RUN = runway, BUR = burrow) and control site (CON) locations within flat-centered polygon (FCP) and high-centered polygon (HCP) tundra near Utqiaġvik, Alaska, USA. Ten replicates of each structure type and 10 control sites were sampled within each habitat type during the summer of 2021.

Supplemental Figure 6. Box plots showing differences, as determined through GLMMs, in a) microbial biomass carbon (MBC), b) microbial biomass nitrogen (MBN), and c) microbial biomass phosphorus (MBP) at brown lemming structures (HAY = hay pile, LAT = latrine, RUN = runway, BUR = burrow) and control site (CON) locations within flat-centered polygon (FCP) and high-centered polygon (HCP) tundra near Utqiaġvik, Alaska, USA. Ten replicates of each structure type and 10 control sites were sampled within each habitat type during the summer of 2021.

Supplemental Figure 7. Box plots showing differences, as determined through GLMMs, in a) β-glucosidase, b) β-cellobiosidase, c) β-xylosidase, d) α-glucosidase, e) LAP, f) NAG, g) Phosphatase, and h) Phosphodiesterase activities (ug MUB g-1 soil hr-1) at brown lemming structures (HAY = hay pile, LAT = latrine, RUN = runway, BUR = burrow) and control site (CON) locations within flat-centered polygon (FCP) and high-centered polygon (HCP) tundra near Utqiaġvik, Alaska, USA. Letters represent statistical differences between structures within habitat sites. Ten replicates of each structure type and 10 control sites were sampled within each habitat type during the summer of 2021.

Supplemental Figure 8. Box plots showing differences, as determined through GLMMs, in soil conductivity at brown lemming structures (HAY = hay pile, LAT = latrine, RUN = runway, BUR = burrow) and control site (CON) locations within flat-centered polygon (FCP) and high-centered polygon (HCP) tundra near Utqiaġvik, Alaska, USA. Letters represent statistical differences between structures within habitat sites. Ten replicates of each structure type and 10 control sites were sampled within each habitat type during the summer of 2021.

Supplemental Figure 1


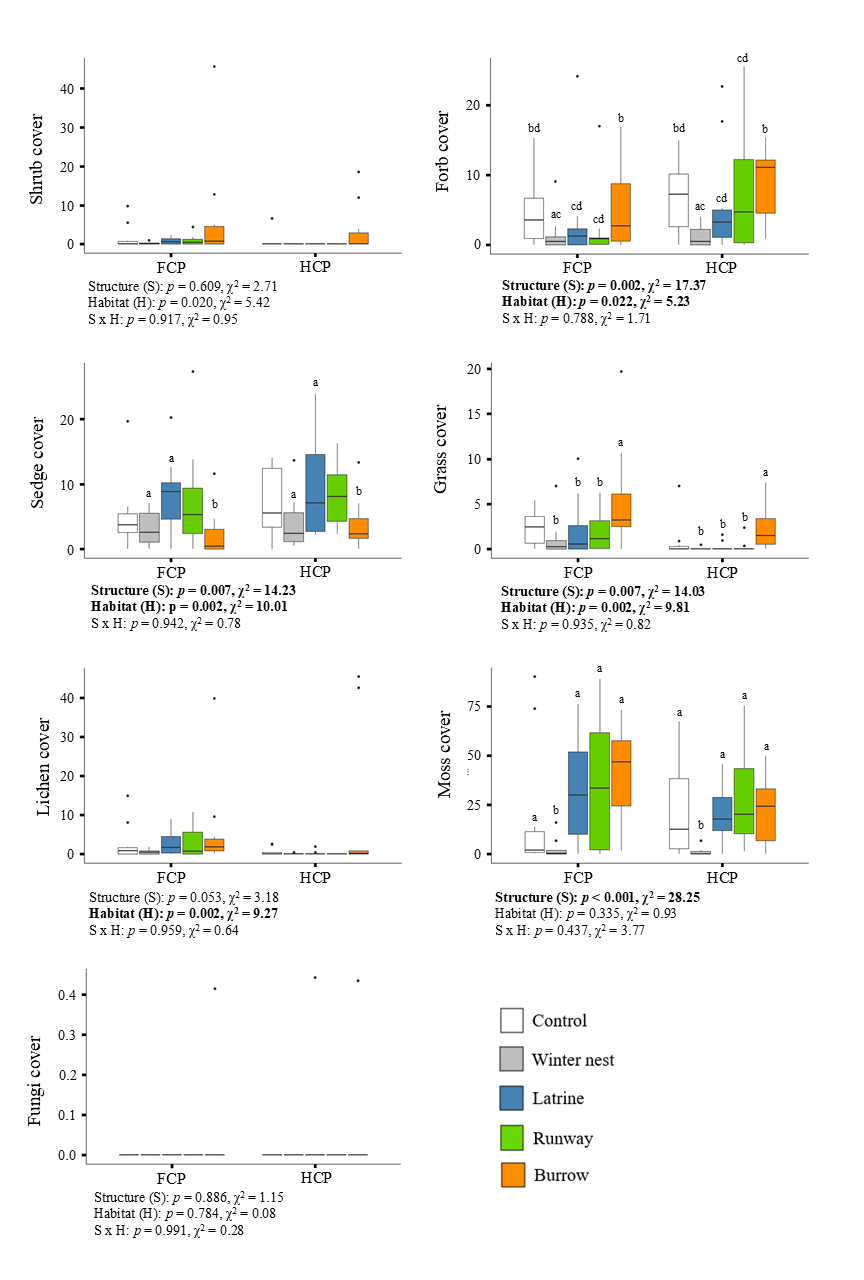


Supplemental Figure 2


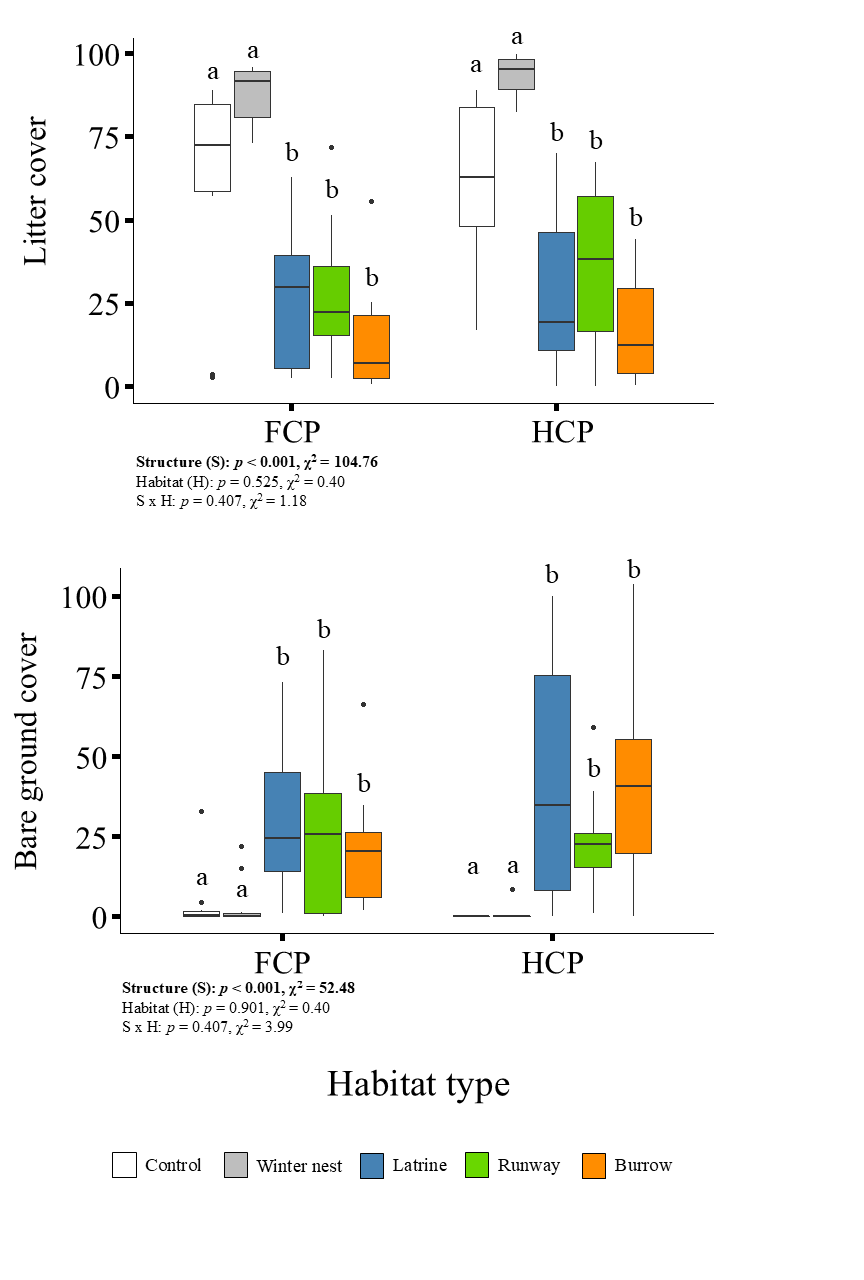


Supplemental Figure 3


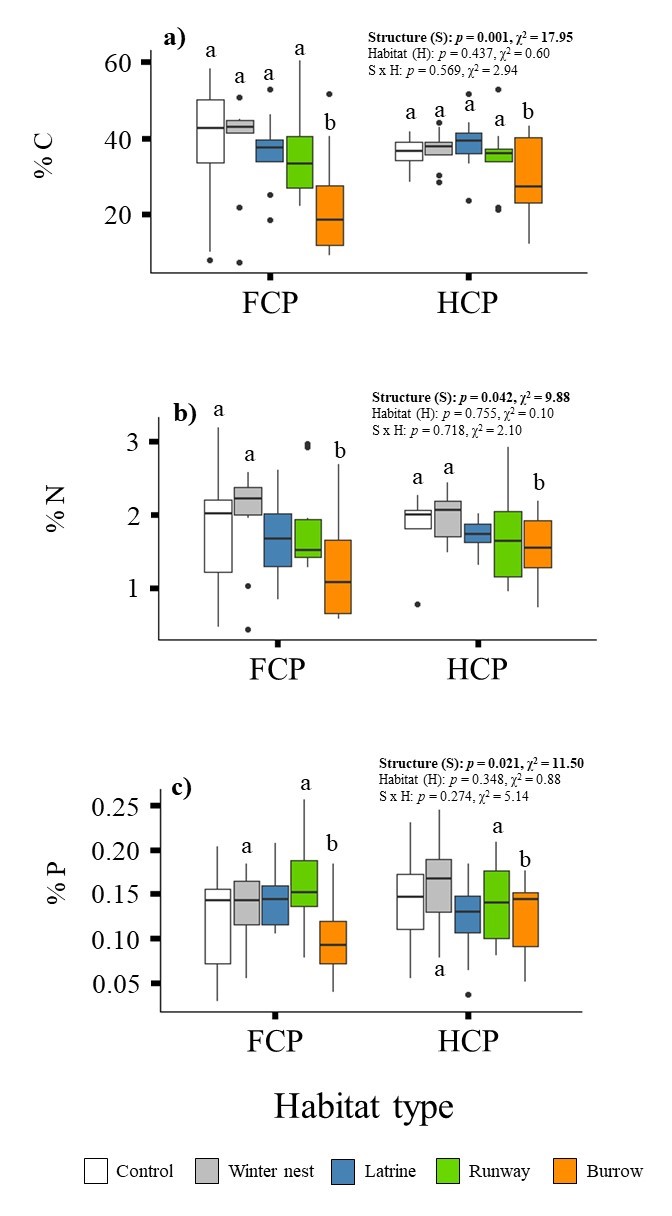


Supplemental Figure 4


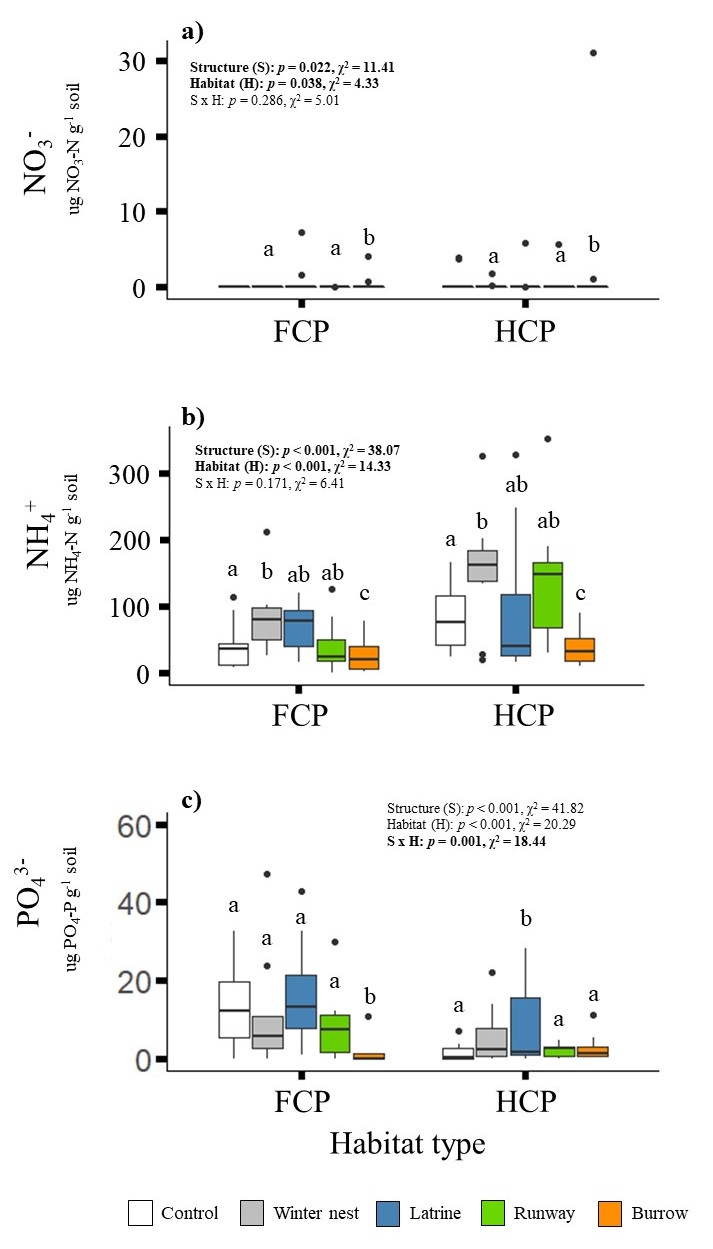


Supplemental Figure 5


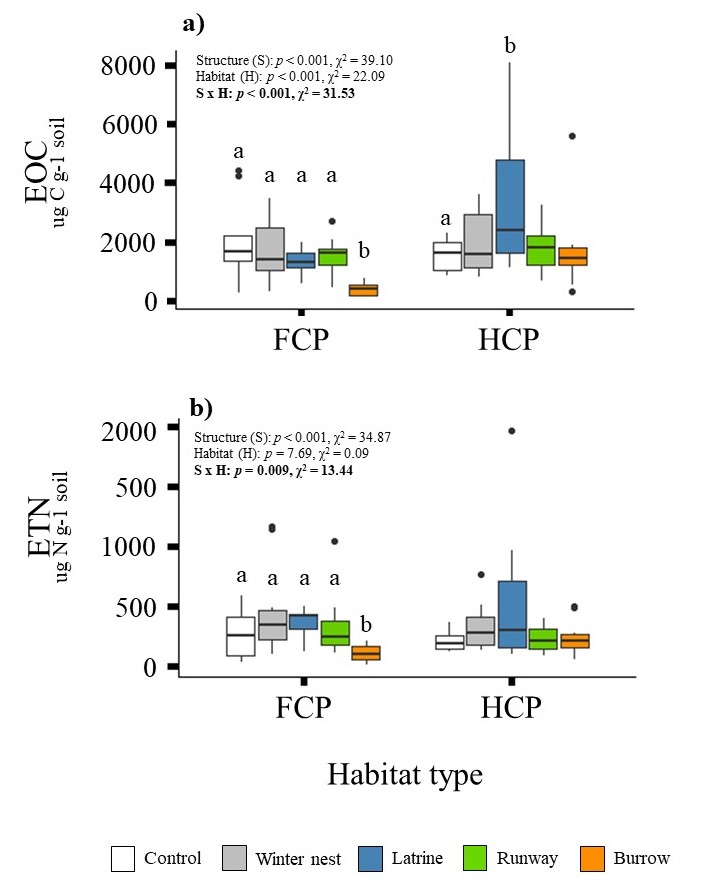


Supplemental Figure 6


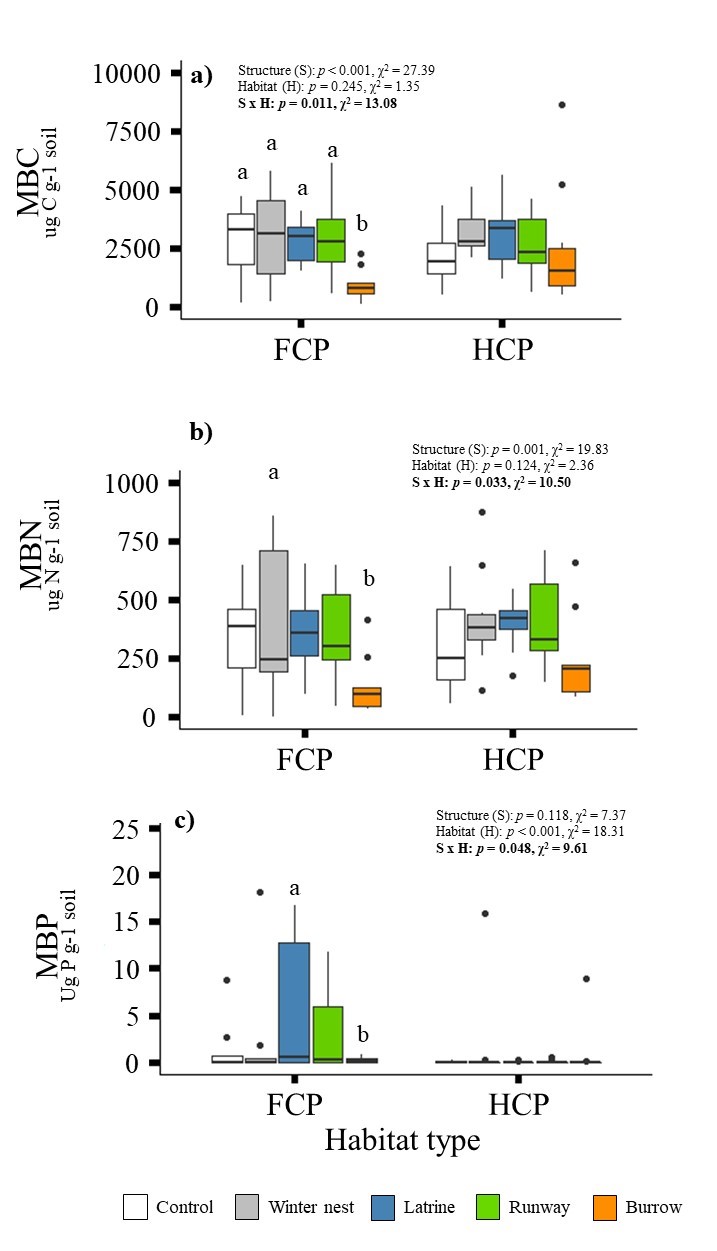


Supplemental Figure 7


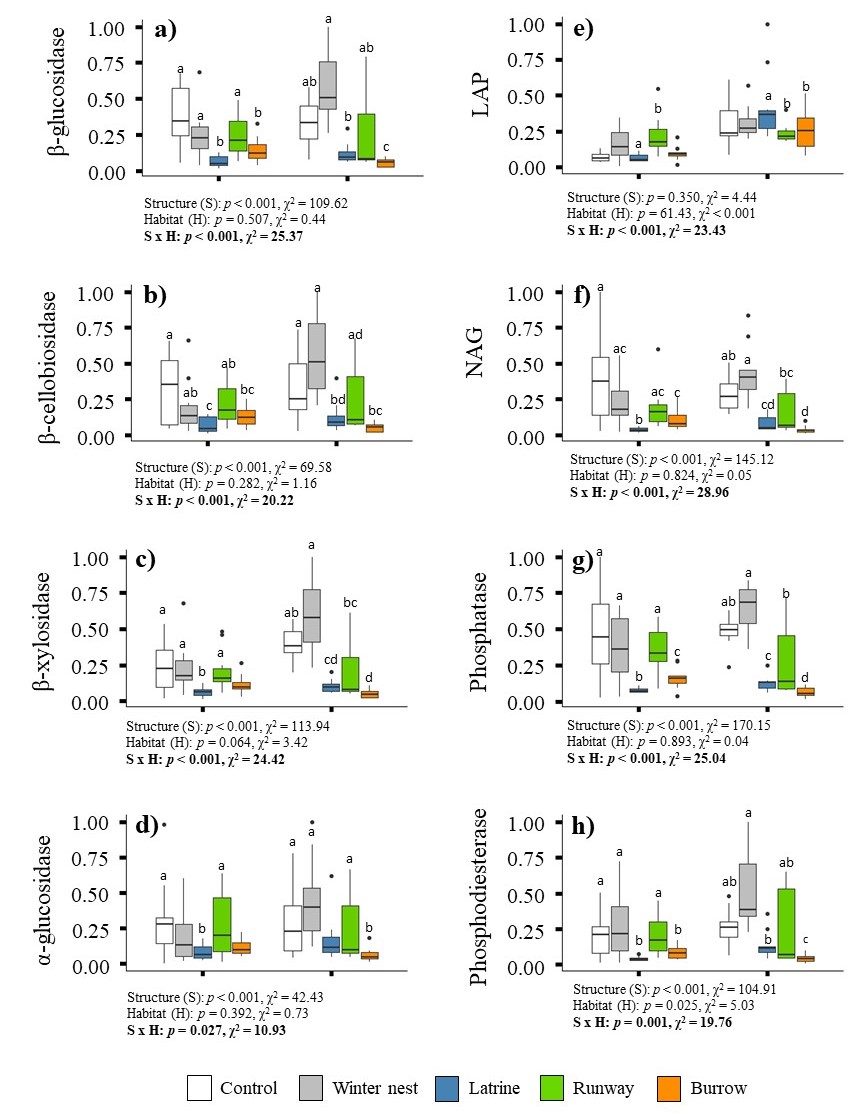


Supplemental Figure 8.


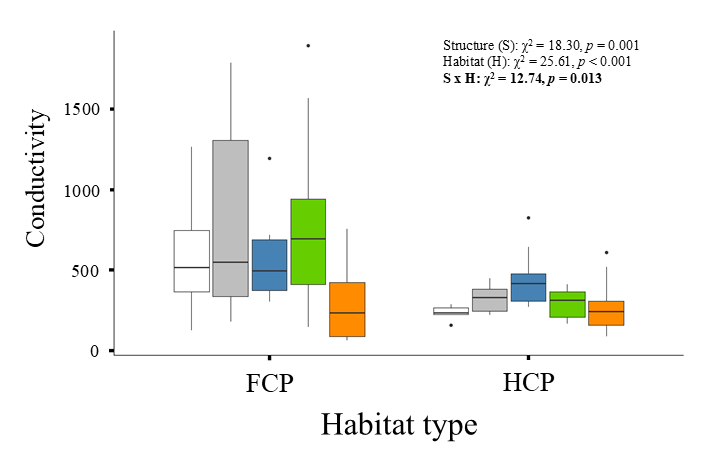

Supplement: Supplementary file 1 — Data S1. [file ECE3-15-e71523-s001.docx]
